# Nanoscale MXene Interlayer and Substrate Adhesion for Lubrication: A Density Functional Theory Study

**DOI:** 10.1021/acsanm.2c01847

**Published:** 2022-08-08

**Authors:** Edoardo Marquis, Michele Cutini, Babak Anasori, Andreas Rosenkranz, Maria Clelia Righi

**Affiliations:** † Department of Physics and Astronomy, Alma Mater Studiorum − University of Bologna, Viale Berti Pichat 6/2, Bologna 40127, Italy; ‡ Department of Mechanical and Energy Engineering, and Integrated Nanosystems Development Institute, Indiana University-Purdue University Indianapolis, Indianapolis, Indiana 46202, United States; § Department of Chemical Engineering, Biotechnology and Materials, University of Chile, Avenida Beaucheff 851, Santiago de Chile 8370456, Chile

**Keywords:** Mxenes, DFT, interlayer adhesion, substrate interaction, solid lubricants, (nano)-tribology

## Abstract

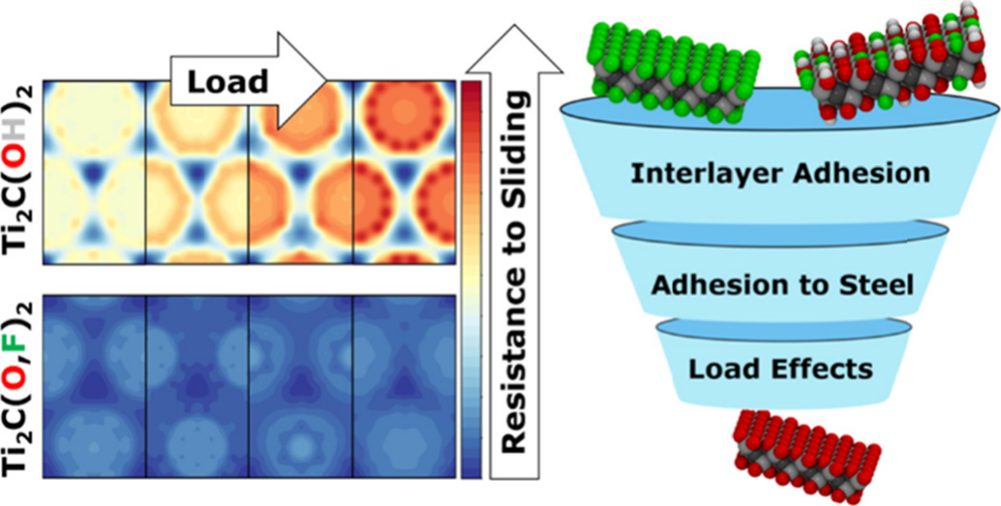

Understanding the interlayer interaction at the nanoscale in two-dimensional (2D) transition metal carbides and nitrides (MXenes) is important to improve their exfoliation/delamination process and application in (nano)-tribology. The layer–substrate interaction is also essential in (nano)-tribology as effective solid lubricants should be resistant against peeling-off during rubbing. Previous computational studies considered MXenes’ interlayer coupling with oversimplified, homogeneous terminations while neglecting the interaction with underlying substrates. In our study, Ti-based MXenes with both homogeneous and mixed terminations are modeled using density functional theory (DFT). An ad hoc modified dispersion correction scheme is used, capable of reproducing the results obtained from a higher level of theory. The nature of the interlayer interactions, comprising van der Waals, dipole–dipole, and hydrogen bonding, is discussed along with the effects of MXene sheet’s thickness and C/N ratio. Our results demonstrate that terminations play a major role in regulating MXenes’ interlayer and substrate adhesion to iron and iron oxide and, therefore, lubrication, which is also affected by an external load. Using graphene and MoS_2_ as established references, we verify that MXenes’ tribological performance as solid lubricants can be significantly improved by avoiding −OH and −F terminations, which can be done by controlling terminations via post-synthesis processing.

## 1 Introduction

From the discovery of two-dimensional (2D) transition metal carbides and nitrides (MXenes) in 2011,^1^ great attention has been devoted to the study of their outstanding performance in several applications such as energy conversion and storage,^2−4^ sensors,^5,6^ electromagnetic shielding,^7,8^ catalysis,^9−11^ and tribology.^4,12−14^ The wide range of technologies, in which MXenes can be employed, originates from the inherent tunability of their chemical composition, which makes them one of the fastest growing 2D materials.^2,15^ MXenes can be described by the general formula M_*n*+1_X_*n*_T_*x*_, where M is an early transition metal (Ti, V, Mo, Cr, Sc, Nb, etc.), X represents carbon and/or nitrogen, *n* can vary from 1 to 3 (high-quality MXenes with *n* = 4 are not easily synthesized),^16^ and T_*x*_ identifies the terminating groups covering the surface (mainly −F, −O, −OH).^2,17,18^ MXenes are synthesized via a top-down synthesis approach from three-dimensional (3D) crystalline MAX precursors with chemical formula M_*n*+1_AX_*n*_ by selectively removing the layers of the A-group elements (mainly group IIIA and IVA of the periodic table) using acidic aqueous solutions.^12,17^ The composition of surface terminations depends on the etching conditions, in particular, the etchant type and concentration, as well as etching temperature and duration. Experimental studies using nuclear magnetic resonance (NMR),^19,20^ X-ray photoelectron spectroscopy (XPS),^21^ and thermal gravimetric analysis coupled with mass spectrometry (TGA-MS)^22^ verified that the MXene surfaces are terminated with a random distribution of fluorine, oxygen, and hydroxyl groups. The capability of weakly interacting, layered 2D materials, such as graphene or molybdenum disulfide, to effectively reduce friction makes MXenes appealing for tribology applications.^23,24^ Indeed, an increasing number of tribological experiments at the macroscale have been carried out over the last 3 years to explore and confirm the potential of MXenes as solid lubricants.^13,25−29^

While Ti_3_C_2_T_*x*_ is by far the most investigated one of all experimentally synthesized MXenes, Ti-based MXenes have been also studied with density functional theory (DFT) calculations and classical molecular dynamics (MD) simulations.^30−34^ Hu et al. investigated the interlayer coupling of Ti_*n*+1_C_*n*_T_2_ (T: OH, O, and F).^30^ They evaluated the binding energies (*B_e_*) of stacked Ti_3_C_2_T_2_ considering both homogeneous and heterogeneous interfaces (e.g., Ti_3_C_2_T_2_@Ti_3_C_2_T’_2_ with T ≠ T’), finding that the *B_e_* of different terminations followed the order Ti_*n*+1_C_*n*_(OH)_2_ > Ti_*n*+1_C_*n*_O_2_ > Ti_*n*+1_C_*n*_F_2_. In subsequent studies,^31,32^ the static friction coefficients for the interlayer sliding of Ti_*n*+1_C_*n*_O_2_ (*n*: 1, 2, and 3) were calculated, thus deriving minimum energy pathways on the potential energy surface (PES). Moreover, in a very recent work, Serles et al.^34^ exploited friction force microscopy (FFM) combined with DFT studies to evaluate the lubricating properties of Ti_3_C_2_T_*x*_ flakes against a diamond-tipped cantilever. They demonstrate that by annealing Ti_3_C_2_T_*x*_ flakes, a reduction of −OH terminations on the surface is achieved in favor of −F and −O, leading to reduced frictional forces.

However, it is important to point out that interlayer interactions for Ti_*n*+1_C_*n*_T_*x*_ MXenes with mixed terminations are yet to be studied from a numerical point of view. We consider this aspect highly relevant because experimental characterization verified a mixture of different surface terminations on MXenes.^20−22,34^ To fill this gap, we exploit DFT calculations to investigate the behavior of Ti-based MXenes with two types of surface terminations combining fluorine and oxygen, fluorine and hydroxyl, as well as hydroxyl and oxygen, considering with stoichiometric ratios of 1:3, 2:2, and 3:1. To unravel the effect of the simultaneous presence of −F, −O, and −OH on the surface, we also model layers of Ti_2_C(F_1/3_,O_1/3_,OH_1/3_)_2_. Furthermore, we investigated the influence of the carbon/nitrogen content and the layer thickness. It is worth pointing out that our models, involving different combinations of terminations, allow us to deeply understand the relationship between composition and interlayer properties at the nanoscale. The stoichiometric ratios considered do not pretend to mimic the composition of a particular case of realistic MXenes.

We first evaluated the interlayer binding energy for homo-, hetero-, and mixed interfaces and then calculated its variation as a function of the relative lateral position of the layers, constructing the PES. The presence of a mixture of elements with different chemical connectivity, electronegativity, and steric hindrance makes the PES much richer in electronic features than in other solid lubricants such as transition metal dichalcogenides and graphene.^35−37^ We evaluated the PES corrugation in the presence of an external normal load applied (ranging from 1 to 10 GPa), demonstrating how the load dependence of the resistance to sliding is governed by the surface termination of MXenes. Finally, because the efficiency of a solid lubricant depends not only on the interlayer interactions but also on the layer–substrate interaction, we investigated the influence of the termination (T) on the MXene adhesion on ferrous substrates, that is, pristine iron and hematite. The analysis at the nanoscale is carried out in comparison with well-established solid lubricants such as MoS_2_ and graphene.^38^ The results of our study indicate the major role of MXenes’ surface terminations in determining their exfoliation ability as well as their (nano)tribological performance with the overall aim to reduce friction and wear.

## 2 Systems and Methods

We performed spin-polarized DFT calculations employing version 6.7 of the Quantum ESPRESSO package.^39^ The generalized gradient approximation (GGA) within the Perdew–Burke–Ernzerhof (PBE) parametrization under the consideration of dispersion interactions was adopted to describe the electronic exchange and correlation.^40^ The electronic wave-functions were expanded on a plane-waves basis that was truncated with a cutoff of 50 Ry. A cutoff of 400 Ry was employed for the charge density. The ionic species were described by ultrasoft pseudopotentials, those of d-metal ions, that is, Ti, Mo, and Fe, have 12, 14, and 16 explicit electrons for an accurate description of interfacial interactions, respectively. For structural optimization, we adopted default criteria for energy and forces convergence, and we used a Gaussian smearing of 0.02 Ry to better describe the electronic state occupation around the Fermi level. We sampled the Brillouin zone of single cells with a 12 × 12 × 1 Monkhorst–Pack grid, while an equivalent sampling was used for larger cells.^41^

We investigated MXenes interacting with hematite using the PBE functional with the Hubbard correction (PBE + *U*).^42^ The *U* value was set to 4.2 eV as suggested by previous adsorption studies on hematite.^43,44^ The spin of d electrons localized on Fe atoms was assigned to have wave-functions with antiferromagnetic character.^45^ We forced Fe d-orbital occupation to ensure that the wave-function converges on nonmetallic electronic states. We considered the (001) surface, which is a stable low-index hematite crystal facet,^46^ exposing a single Fe atom as termination. We ensured full convergence of the Brillouin zone sampling by using a 6 × 6 × 1 grid of *k* points. For iron, we considered the most stable low-index Fe surface, that is, (110).^47^ In matching the 2D materials with the substrate, we allowed for a maximum deformation of 5% of the unit cell of the 2D materials. To identify the most favorable lateral position of the MXene layer on the complex surface of hematite, we calculated the PES at fixed atomic positions and then relaxed the system in the PES minimum (Figure S9 of the Supporting Information). To avoid spurious interactions, all surfaces and interfaces were built with at least 15 Å of vacuum between vertical replicas.

To account for dispersion interactions, we initially considered several correction schemes, such as the Grimme’s D2^48^ and D3-BJ parametrizations,^49^ the Tkatchenko–Scheffler with iterative Hirshfeld partitioning (TS-H),^50^ many body dispersion (MBD),^51^ dDsC,^52^ as well as vdW-DF2^53^ SCAN functionals,^54^ in which dispersion forces are included directly into the density functional. We compared the results with high-level theory methods, such as the random phase approximation (RPA)^55^ and the second-order Møller–Plesset perturbation theory (MP2)^56^ from our previous work.^57^ The scheme adopted in this work consisted of an ad hoc version of the D2 parametrization, referred to as D2_NG_, in which the C_6_ coefficient and the van der Waals radius *R*_0_ of the metal atoms (titanium and iron) are replaced with those of the preceding noble gas, that is, argon. This approach demonstrated to give good results for similar 2D materials.^57^ In particular, for the C_6_ coefficient (in units of J nm^6^ mol^–1^), we used a value of 4.61 instead of 10.80, while for the van der Waals radius *R*_0_ (in Å), we used 1.595 instead of 1.562. All simulations related to the influence of the dispersion forces (Section 3.1) were carried out using the Vienna Ab initio Simulation Package (VASP) code.^58^ For more detailed information regarding the simulations conducted, please refer to the Supporting Information (SI).

**Figure 1:**
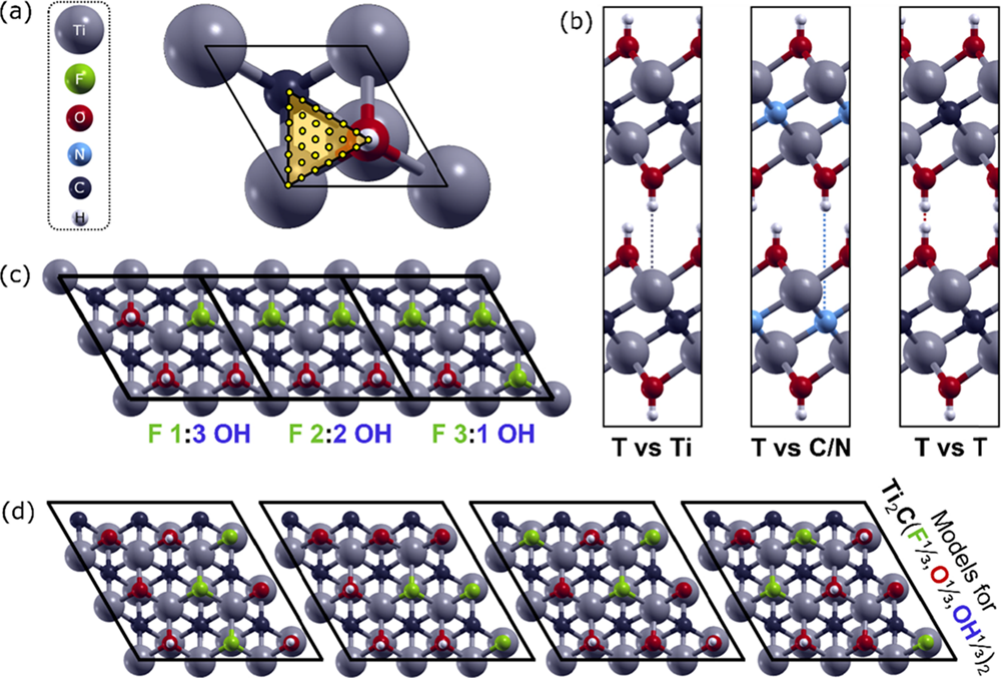
(a) Top-view of the hexagonal cell employed for MXenes with single-type terminations. Lateral displacements considered for the construction of the potential energy surfaces are also reported. The grid of points is then replicated using symmetry operators to fill the cell homogeneously. (b) Lateral view of the relative lateral positions with high symmetry, where the terminations (T) point toward Ti (left), C/N (middle), or toward each other (right). (c) Top-view of the unit cells employed for MXenes with two different types of termination on the surface. Ti_2_C(F_1/4_OH_3/4_)_2_, Ti_2_C(F_1/2_OH_1/2_)_2_, and Ti_2_C(F_3/4_OH_1/4_)_2_ are exemplarily shown with a compact representation, but they have been considered individually for the calculations. (d) Top views of the unit cells employed to model MXene surfaces simultaneously covered by −F, −O, and −OH, that is, Ti_2_C(F_1/3_,O_1/3_,OH_1/3_)_2_. All surfaces have the same chemical composition (T_*x*_: −F, −O, and −OH in the ratio 3:3:3) but differ in the relative atomic position of the terminations with respect to M and C atomic sites.

To model MXene layers, we considered both single-species terminations and mixed terminations that include different passivating species. The interfaces obtained by stacking two MXenes layers will be referred according to the mating surfaces: a “homo-interface” (“hetero-interface”) is formed by two identical (different) MXenes with single-species terminations, for example, Ti_2_CF_2_@Ti_2_CF_2_ (Ti_2_CF_2_@Ti_2_CO_2_). In contrast, a “mixed-interface” is composed of two MXenes covered by two or three different types of terminations (e.g., Ti_2_C(F_1/4_OH_3/4_)_2_@Ti_2_C(F_1/4_OH_3/4_)_2_ or Ti_2_C(F_1/3_,O_1/3_,OH_1/3_)_2_@Ti_2_C(F_1/3_,O_1/3_,OH_1/3_)_2_). MXene layers with single-type terminations were modeled with hexagonal cells as depicted in Figure 1a. The equilibrium value of the lattice parameter “*a*” of the cell was derived following the procedure reported in Figure S1. To identify the most favorable stacking of parallel layers, we considered the high-symmetry lateral positions represented in Figure 1b. In these three configurations, the atom or group, belonging to the termination (T) of the upper layer is placed on top of the metal atom (T versus Ti), the carbon/nitrogen (T versus C/N), or another surface termination (T versus T) of the bottom layer. MXene layers with mixed terminations were modeled employing double- or triple-sized hexagonal cells. This increase in cell dimensions was necessary to investigate different stoichiometric ratios of the surface terminations. Figure 1c exemplarily depicts the view of Ti_2_C(F_1/4_OH_3/4_)_2_, Ti_2_C(F_1/2_OH_1/2_)_2_, and Ti_2_C(F_3/4_OH_1/4_)_2_, while Figure 1d shows four different isomers of Ti_2_C(F_1/3_,O_1/3_,OH_1/3_)_2_, which differ in the relative atomic position of the terminations. In the case of mixed interfaces, the number of high-symmetry lateral positions considered for identifying the most stable stacking was increased (please refer to the Supporting Information Figure S2 for more details). For these calculations, no constraints were imposed, that is, no atom was fixed during relaxation. The partial atomic charges for stacked and adsorbed MXenes were evaluated by means of the Bader Charge analysis.^59^

For both homogeneous and heterogeneous interfaces, we constructed the PES experienced by the upper monolayer upon translation above the lower one. Because of the presence of several species with different chemical natures, we increased the number of relative lateral positions (*x*, *y*) to capture all features of the PES. For each lateral displacement, the *x* and *y* atomic coordinates were kept fixed, while the *z* coordinate was relaxed so that the equilibrium interfacial distance was reached for every lateral position. Figure 1a reveals the grid of points used to calculate the PES, which belongs to the irreducible zone of the hexagonal cell. To investigate the effect of increasing normal loads, we repeated the calculation of adhesion in the presence of a force perpendicular to the basal plane and applied to the highest Ti atom of the top layer. In this case, the lower Ti atom of the bottom layer was fixed during relaxation. We also verified that the equilibrium value of the lattice parameter “*a*” of the cell was not affected by the presence of an external load (Figure S1).

## 3 Results and Discussion

### 3.1 Dispersion Correction

**Figure 2:**
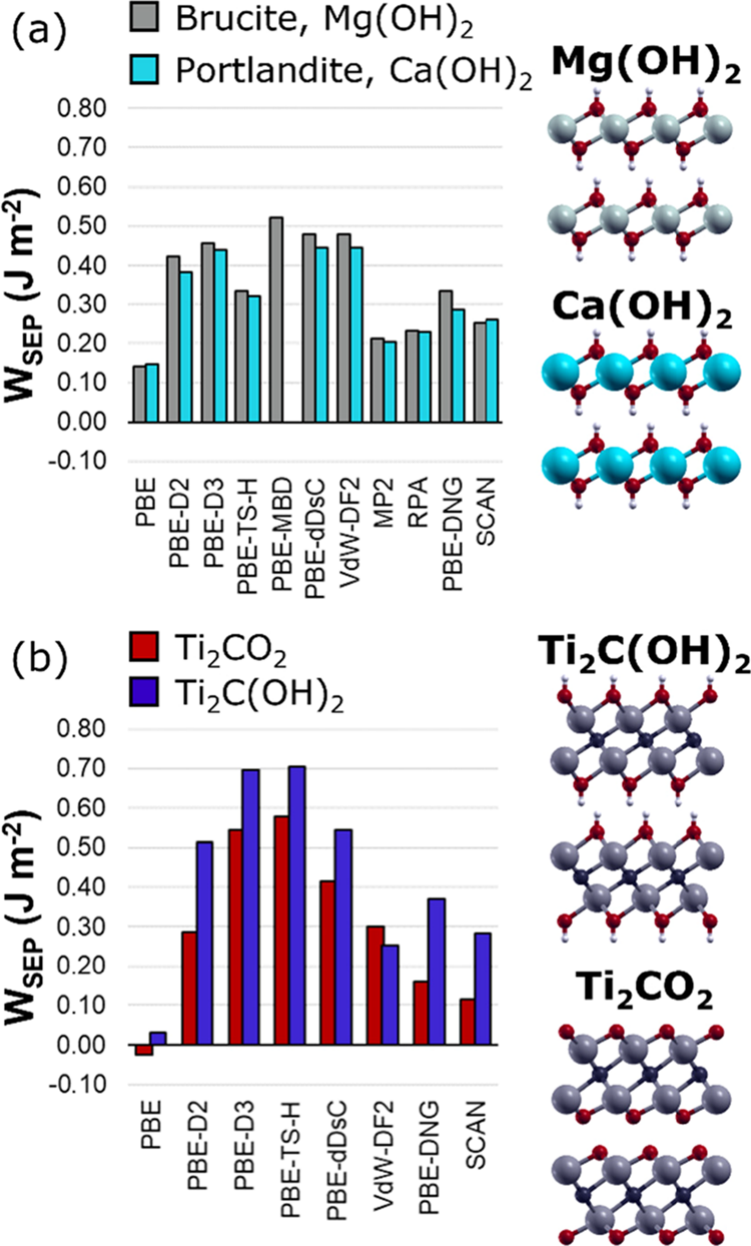
Work of separation for (a) brucite (gray), portlandite (light blue), (b) Ti_2_C(OH)_2_ (blue), and Ti_2_CO_2_ (red) calculated with different dispersion correction methods. PBE-DNG refers to the D2 scheme with the Ti parameters replaced by those belonging to Ar.

In 2D inorganic materials, as investigated in this work, the interlayer interactions comprise H-bonding, dipole–dipole, and dispersion London interactions.^57^ Dispersion forces are neglected by most of plain DFT functionals, and in the last two decades, several approaches have emerged to overcome this limitation.^60^ However, most of the available dispersion correction methods for DFT have been developed for organic molecules. Although they have been holistically tested for periodic organic systems, such as polymers^61−63^ and molecular crystals,^64^ they should be carefully applied to inorganic solid-state materials.^57^ More advanced, parameter-free methodologies such as the Møller–Plesset perturbation theory (MP2) and the random phase approximation (RPA) can capture the elusive dispersion forces in an accurate way in solid inorganic systems. These methods are computationally too demanding to be employed for a systematic study as presented in this study. However, they can be used as a benchmark for the proper choice of the parameters in DFT schemes, which include the dispersion interactions in a parametric way. Unfortunately, a direct application of the abovementioned fully ab initio methods to conductive materials with a complex electronic structure as MXenes is not straightforward. In this regard, insulating materials, which have similar structures as MXenes, can be employed as more feasible test cases. Materials with these features are natural clays, such as Mg and Ca hydroxides. They have the same octahedral metal coordination of MXenes but an insulating electronic structure (Figure 2a). For these materials, we have computed accurate MP2 and RPA work of separation, thus comparing the results with the most common dispersion-corrected DFT functionals available for solid-state materials. The results clearly indicate that the choice of the dispersion scheme is crucial to obtain accurate adhesion energies. Grimme’s D2, D3, TS-H, and MBD a posteriori corrections overbind Mg and Ca hydroxides layers, almost doubling the interaction energy. Similar results are obtained with the vdW-DF2 functional, which has been employed to study the tribological properties of MXenes, as shown in previous studies.^31,32^ Interestingly, SCAN functional gives remarkably accurate results. The drawback of the SCAN functional, belonging to the meta-GGA family, relates to its computational cost, which is roughly eight times higher than the GGA PBE functional.

Aiming at finding a fast and accurate approach to investigate MXenes systematically, we have also tested the -D_NG_ a posteriori correction. This approach applies the pairwise Grimme’s -D2 scheme with the difference that the atomic parameters employed to describe the metal atoms are replaced with those of the proceeding noble gas. This is done to reduce the dispersion energy coming from metals atoms, whose standard parameters better describe a neutral isolated atom than a metal atom within a network of covalent-ionic bonds. Indeed, in this framework, the metal atom has lowered atomic polarizability because of the positive charge localized on the atom. A similar idea has been implemented in the very recent Grimme’s D4 scheme,^65^ which is nowadays unavailable in the Quantum ESPRESSO suite. The computed adhesion energy for Mg and Ca hydroxides indicates that PBE-D_NG_ is a fast and accurate methodology for computing interlayer energy for MXene-type model materials.

After tuning the parameters of the PBE-D_NG_ scheme considering Mg and Ca hydroxides as a benchmark, we extended the method toward Ti_2_C(OH)_2_ and Ti_2_CO_2_ MXenes (Figure 2b). In this case, MP2 and RPA methods cannot be applied straightforwardly. Consequently, we have considered the SCAN functional results as the reference method because of the good results obtained for Mg and Ca hydroxides. Furthermore, the SCAN functional can compute accurately dispersive interactions regardless of the material electronic structure, that is, conductive or insulating.^65^ The results for MXenes agree well with the previous analysis: the PBE-D_NG_ approach is the only method capable of reproducing, with fair accuracy, both the overall absolute adhesion values and the order of stability of the SCAN reference method. These results indicate that the PBE-D_NG_ method is the most suitable approach for studying MXenes’ interlayer interaction.

Following the analysis for the MXene-MXene interface, we employ the PBE-D_NG_ scheme for describing hematite systems. Indeed, iron atoms are covalently bonded to oxygen and have a positive charge localized on Fe atoms. In contrast, pristine iron has metallic-type bonds. Consequently, we checked the effect of using -D_NG_ and parameters for the Fe atom compared to standard -D2 on the adsorption of 2D materials (see Figure S8). Our results indicate that the use of -D_NG_ dampens the interfacial interaction with respect to -D2, while giving the same trend of *W*_SEP_. Therefore, we employed the PBE-D_NG_ method for pristine iron to have an equivalent description of dispersion forces.

Interestingly, the vdw-DF2 functional indicates that the O termination induces higher *W*_SEP_ than the OH-termination in homogeneous interfaces, which disagrees with the reference method, that is, SCAN, and all the other functionals employed in this work. Our result suggests that by employing the vdw-DF2 functional,^31^ spurious results can be obtained if the interfacial properties of MXenes are compared for different types of terminations.

### 3.2 Interlayer Adhesion

**Figure 3:**
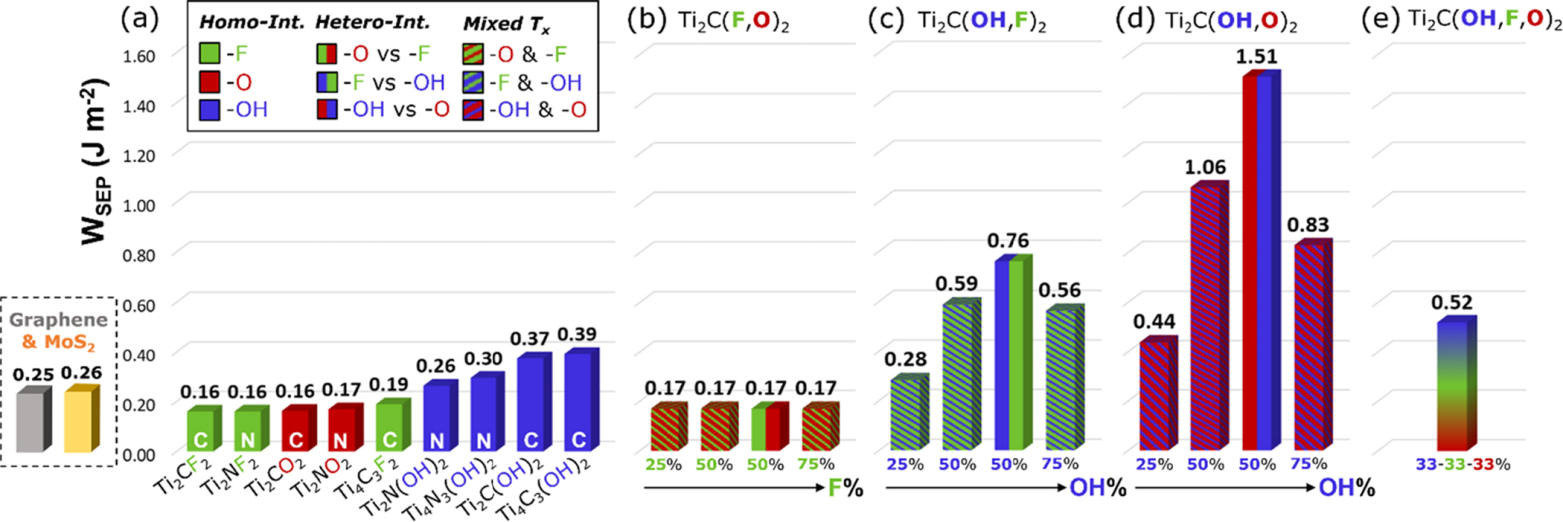
Work of separation W_SEP_ for (a) homo-interfaces with single-type terminations (in solid color bars), (b–d) mixed interfaces combining different termination pairs (thin oblique lines motif) and hetero-interfaces (vertical-line pattern). e) Average value of *W*_SEP_ for Ti_2_C(F_1/3_,O_1/3_,OH_1/3_)_2_@Ti_2_C(F_1/3_,O_1/3_,OH_1/3_)_2_ interfaces considered. The *W*_SEP_ values for graphene and MoS_2_ bilayers are provided as references.

In Figure 3a, we report the energies required to separate homogeneously terminated Ti_*n*+1_X_*n*_T_*x*_ bilayers (*n*:1 and 3; X: C and N; T_*x*_: F, O, and OH). Schematics of MXene bilayers are shown in Figure 2b. The results are sorted starting with the lowest energy configurations. The work of separation (*W*_SEP_), the opposite of the adhesion energy (*E*_ADH_), is obtained as follows:
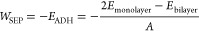
1where *A* is the contact area. The values of *W*_SEP_ obtained for Ti_2_CF_2_/Ti_2_CO_2_ and Ti_2_NF_2_/Ti_2_NO_2_ indicate that no differences occur between −F- and −O-terminated MXenes (*W*_SEP_ ≈ 0.16 J m^–2^). The similar behavior relates to the chemical similarities of the terminating atoms, which both possess high electronegativity. When terminated with −OH, homo-interfaces of Ti_2_C(OH)_2_ (*W*_SEP_ = 0.37 J m^–2^) and Ti_2_N(OH)_2_ (*W*_SEP_ = 0.26 J m^–2^) show higher values of work of separation. The increased values of *W*_SEP_ obtained when moving from −F or −O to −OH-terminated MXenes are consistent with the results of Hu et al.^30^

In general, our calculations indicate that the substitution of carbon by nitrogen does not change the extent of the interaction when MXenes are F- or O-terminated, implying that the C/N ratio does not notably affect the interfacial properties for F/O-terminated MXenes. However, the changes become more noticeable for OH-terminated MXenes, for instance, Ti_4_C_3_(OH)_2_ with *W*_SEP_ = 0.39 J m^–2^, which is higher than that of Ti_4_N_3_(OH)_2_ with *W*_SEP_ = 0.30 J m^–2^. Our results demonstrate that, in the presence of hydroxyl groups, carbides tend to interact more than nitrides.

Finally, it is worth noting that no remarkable differences stand out when comparing thin MXenes (Ti_2_XT_*x*_) with thicker ones (Ti_4_X_3_T_*x*_). This suggests that the interaction between layers is mainly governed by the surface terminations of the outer layer, which are barely modified by increasing the thickness. However, thicker MXenes systematically show slightly higher *W*_SEP_ values (by 0.02/0.04 J m^–2^), as the number of atoms interacting through long-range dispersion forces increases.

Figure 3b–d reports the cases in which two terminations are simultaneously present at the interface of thin MXene bilayers (*n:* 1, carbides: X: C). The bars with the thin oblique lines pattern show the work of separation for mixed interfaces composed of two identical MXenes each covered with two different terminations. The two colors of the thin lines follow the same color code employed for the homo-interfaces: green, red, and blue highlight the presence of −F, −O, and −OH terminations, respectively. We investigated all combinations between termination pairs: −F and −O (Figure 3b), −OH and −F (Figure 3c), and −OH and −O (Figure 3d) with different coverages ranging between 25 and 75% for each termination group. The bars with the vertical-line pattern refer to hetero-interfaces, which are composed of two MXene layers, each terminated with a different type of termination. The values of *W*_SEP_ presented in Figure 3b are not influenced by the ratio between −F and −O as *W*_SEP_ is always equal to 0.17 J m^–2^, which is almost the same value as previously presented for the −F- and −O-terminated homogeneous interfaces (Figure 3a). This result suggests that fluorine and oxygen confer almost the same properties to the bilayer. However, the presence of hydroxyl groups at the interface considerably increases *W*_SEP_ (Figure 3c, d). When a fully −OH-terminated MXene is coupled with a fully −F- or −O-terminated surfaces, the interaction is maximized (*W*_SEP_ = 0.76 J m^–2^ or *W*_SEP_ = 1.51 J m^–2^). Even for MXenes with mixed T_*x*_ (−OH and −F, or −OH and −O), the interaction is much stronger compared to the homogeneous interfaces. Interestingly, *W*_SEP_ does not increase linearly with the −OH percentage but shows a maximum for a coverage of 50%. At 50% −OH coverage, all hydroxyl groups can establish hydrogen bonds with the involved fluorine or oxygen atoms in an on-top configuration. However, when there is a lack of −OH terminations (below 50% −OH coverage), the number of hydrogen bonds formed at the interface is reduced. With an excess of −OH terminations (above 50% −OH coverage), the interaction is reduced because of the unavoidable steric hindrance of OH–HO stacking (please refer to Figure S3 in the Supporting Information).

The bar with fuzzy colors in Figure 3e refers to fully mixed interfaces, where the mated MXene layers are both passivated with −F, −O, and −OH (i.e., Ti_2_C(F_1/3_,O_1/3_,OH_1/3_)_2_@Ti_2_C(F_1/3_,O_1/3_,OH_1/3_)_2_). As mentioned in the Section 2, four isomers were examined to model the fully mixed layers. The four isomers of Ti_2_C(F_1/3_,O_1/3_,OH_1/3_)_2_ differ from each other regarding the relative position of the terminations, while maintaining the overall chemical composition. *W*_SEP_ = 0.52 J m^–2^ represents the average value obtained by stacking the four isomers considered. Interestingly, the *W*_SEP_ values are very similar, ranging between 0.51 and 0.54 J m^–2^ (with a standard deviation of 0.01 J m^–2^). This implies that the interaction between two “realistic” MXene layers does not depend on the relative position of the surface terminations, but only on their chemical composition. In Figure S3, we depict the fully mixed interfaces after relaxation, which are governed by hydrogen bond interactions between −OH (donors) and −O or −F (acceptors) groups. Even for MXenes with mixed terminations, we confirm that the interaction between layers is mainly driven by the concentration of hydroxyl groups on the surface.

**Figure 4:**
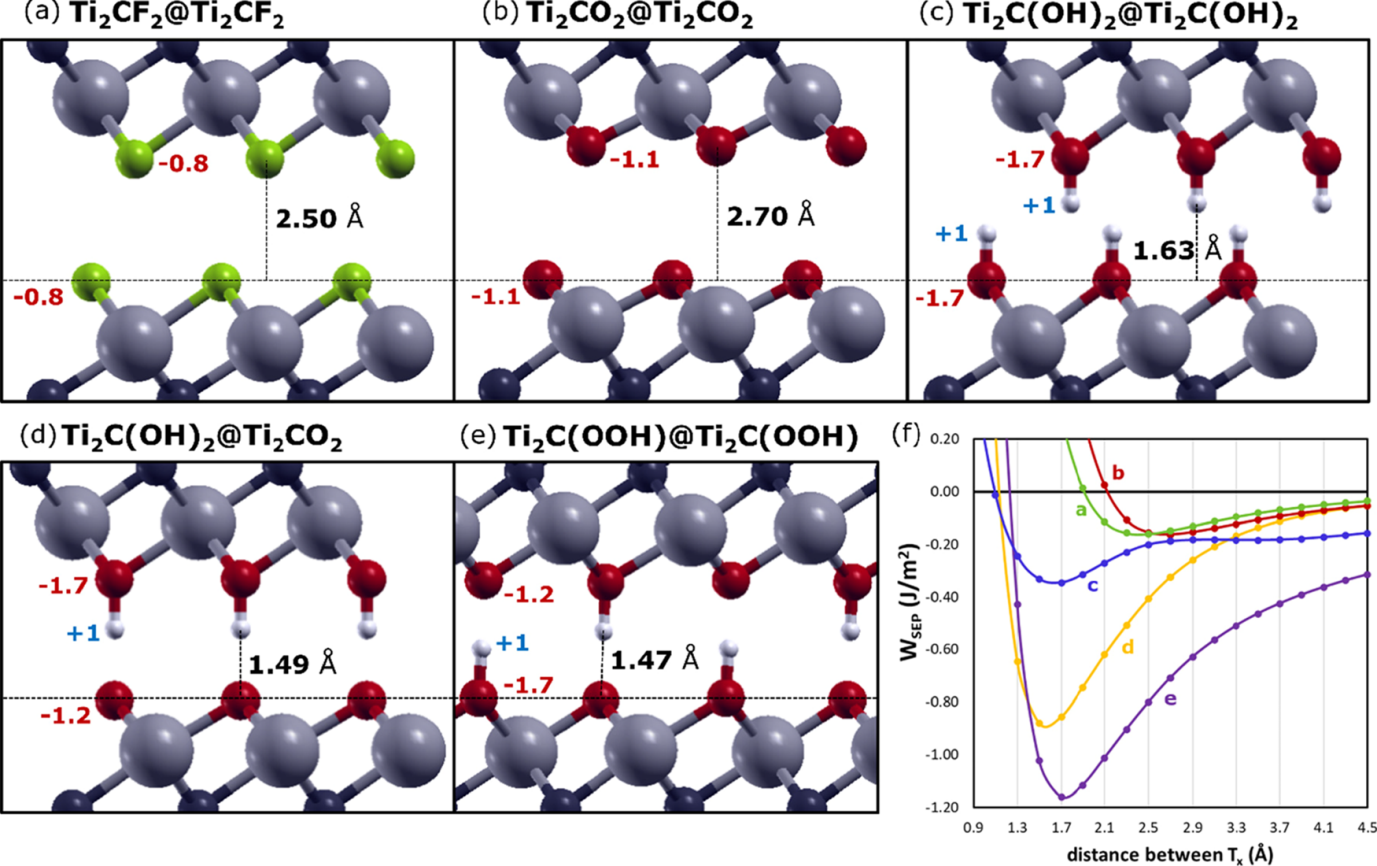
Optimized configurations of stacked MXenes with equilibrium distances and partial atomic charges of the terminations (expressed as a fraction of elementary charge unit “e”). (a–c) Ti_2_CT_*x*_@Ti_2_CT_*x*_ interfaces with homogeneous terminations having different terminations including T_*x*_ = F or O or OH. (d) refers to the heterogeneous interface Ti_2_C(OH)_2_@Ti_2_CO_2_, while (e) shows the pairing of MXenes with mixed O and OH terminations (Ti_2_COOH@Ti_2_COOH). The partial charges of the innermost atoms range between (+1.6e) – (+2.0e) for Ti and (−1.9e) – (−1.7e) for C, depending on the electronegativity of the termination. (f) Summary of the perpendicular potential energy surfaces (pPES) for all systems considered.

In Figure 4, the optimized configurations of MXenes bilayers are reported, along with the equilibrium distances and partial atomic charges on the terminations (red and blue numbers). We also provide the perpendicular potential energy surfaces (pPES), which are obtained by calculating the adhesion energy between the paired surfaces at different fixed distances. Ti_2_CF_2_ (Figure 4a) and Ti_2_CO_2_ (Figure 4b) bilayers are characterized by terminations with high electronegativity, thus presenting negative partial charges. The equilibrium interlayer distance reflects the magnitude of the partial negative charge of the termination. This implies that the electrostatic repulsion governs the properties of the interface for F- and O-terminated MXenes. Indeed, for these systems, the dispersion forces are essential to bind two MXene layers. In Figure S4, we show that by “turning off” the D2 dispersion correction during calculation, both layers move away to infinite distance.

The presence of −OH terminations induce a further dipole–dipole interaction between the layer terminations, which is not present for −O and −F terminations (Figure 4c). This additional attractive interaction moves both MXene layers closer to each other, while increasing *W*_SEP_. In Figure 4d, e, the schematics of hetero- and mixed interfaces with −OH and −O terminations are shown. The explanation for the higher *W*_SEP_ calculated for these systems lies in the formation of hydrogen bonds between the hydroxyl group (H-bond donor) and oxygen atoms (H-bond acceptor), leading to a reduced interlayer distance of about 1.5 Å. The presence of H-bonds is also confirmed by the increase in the negative partial charge of oxygen atoms acting as H-bond acceptors (−1.2*e* in Figure 4d, e instead of −1.1*e* observed for the Ti_2_CO_2_ bilayer in Figure 4b). In Figure 4f, we reported *W*_SEP_ as a function of the interlayer spacing. It becomes evident that the termination controls the nature of the layer attraction from pure dispersive (Figure 4a, b) to dipole–dipole (Figure 4c) and hydrogen-bonding (Figure 4d, e) interactions.

Our results point toward the relevance of mixed terminations, which have not been considered in previous computational studies of MXenes’ tribology. Li et al. measured the adhesion energy between Ti_2_CT_*x*_ bilayers with atomic force microscopy (AFM),^66^ finding *W*_SEP_ of about 0.6 J m^–2^. This experimental value can only be compared to the average *W*_SEP_ calculated for MXenes with mixed surface terminations, that is, 0.52 J m^–2^. The slight difference between the experimental and calculated values probably relates to vacancy defects, which are not considered in our models, although being present in realistic surfaces.^67^ Moreover, our calculations indicate that the interaction between MXene layers can be tailored by reducing the −OH concentration on the surface. The respective interaction can be weakened down to values that are lower than those obtained for well-established solid lubricants such as graphene and MoS_2_ (Figure 3a). We anticipate that this is a critical finding as the control of the distribution of terminations during synthesis and postsynthesis treatments would mark a turning point in the application of MXenes for (nano)-tribological applications.

### 3.3 PES Corrugation

**Figure 5:**
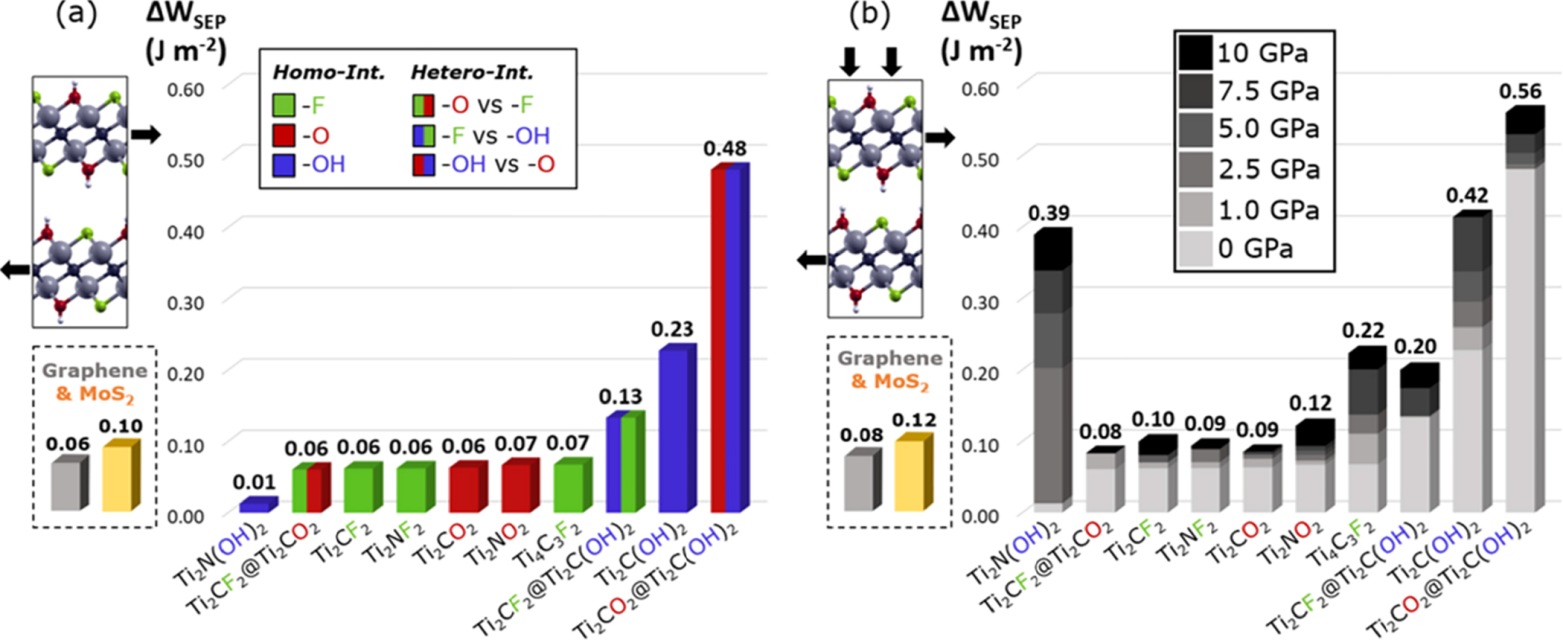
(a) Potential corrugation values for homogeneous (solid color bars) and heterogeneous (vertical-line pattern) interfaces. Green refers to MXenes with −F terminations, red stands for −O, and blue represents −OH. (b) Potential corrugation growth as a function of the normal load applied (the gray-scale coding reflects the applied load with a maximum of 10 GPa). Δ*W*_SEP_ values for graphene and MoS_2_ bilayers are presented for normal loads of 0 and 10 GPa, respectively.

Figure 5a shows the potential corrugation Δ*W*_SEP_ for homogeneous and heterogeneous MXene interfaces without any external load applied. In this regard, Δ*W*_SEP_ represents the maximum energy barrier that needs to be overcome during sliding of two adjacent MXene layers. The potential corrugation is evaluated as the difference between the maximum and minimum *W*_SEP_ experienced during the relative lateral displacement (i.e., Δ*W*_SEP_ = *W*^max^ – *W*^min^).

Concerning homo-interfaces, F- and O-terminated MXenes have similar PESs with a low potential corrugation, Δ*W*_SEP_, of about 0.06–0.07 J m^–2^, which is as low as the corrugation of graphene and lower than that of MoS_2_ bilayers. No difference can be observed between Ti_2_CT_*x*_ and Ti_2_NT_*x*_ for T_*x*_ being −F or −O, as previously observed for *W*_SEP_. Conversely, the potential corrugation for bilayers containing only −OH groups depend on the C/N ratio. At 0.01 J m^–2^, Ti_2_N(OH)_2_ has the lowest potential corrugation among all MXenes and is significantly lower than that of the Ti_2_C(OH)_2_ at 0.23 J m^–2^.

The bars with the vertical-line pattern in Figure 5a refer to hetero-interfaces. We notice that the combined presence of −OH with −F/–O increases the PES corrugation values. The high potential corrugation observed for Ti_2_CO_2_@Ti_2_C(OH)_2_ (0.48 J m^–2^) is consistent with the strong directionality of the hydrogen bonding interaction (unlike dispersive forces). In this regard, to make the layers sliding, all the hydrogen bonds at the interface must be completely broken to induce sliding of the adjacent layer before being reformed, thus generating high energetic barriers. This also happens for Ti_2_CF_2_@Ti_2_C(OH)_2_, but as the interaction between −OH and −F is weaker, the energetic barrier is lower (0.13 J m^–2^).

Figure 5b shows the variation of the potential corrugation as a function of the normal load applied to the upper slab of the MXene bilayer. Bars are organized from left to right based on the Δ*W*_SEP_ value at 0 GPa, and every increment is shown on a gray scale. Because of compressive forces, the corrugations increase with load, which is more consistently seen for the bilayers containing −OH groups at the interface. For loads above 2.5 GPa, the behavior of Ti_2_N(OH)_2_ gets closer to its carbon-based analogue Ti_2_C(OH)_2_. Among F- and O-terminated MXenes, Ti_4_C_3_F_2_ is the only candidate, for which the energy barrier increases with pressure, whereas the thinner bilayers keep their Δ*W*_SEP_ values almost constant, which aligns well with the findings for graphene and MoS_2_.

**Figure 6:**
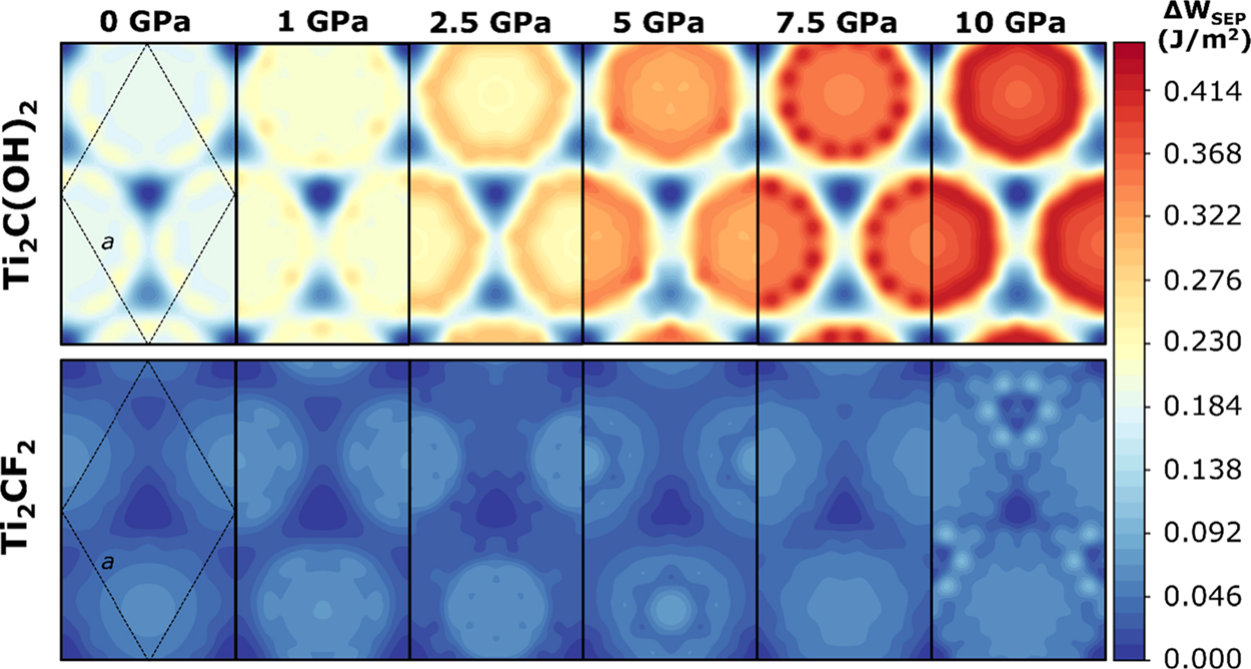
Potential energy surfaces for the sliding motion of Ti_2_C(OH)_2_ (above) and Ti_2_CF_2_ (below) bilayers from zero to 10 GPa load. The color scale is the same for both MXenes. The hexagonal unit cell is schematically shown with black lines in the panels on the very left-hand side.

Finally, Figure 6 demonstrates the PES experienced during sliding for two homogeneous bilayers: Ti_2_C(OH)_2_ and Ti_2_CF_2_. For Ti_2_C(OH)_2_, a color change toward red color becomes visible when moving at higher loads. This clearly implies an increase in the potential corrugation with the load. In contrast, the external pressure does not induce large variations in the corrugation for Ti_2_CF_2_. However, it is worth mentioning that minor electronic effects on the PES motif can be seen. Further charge density analysis is necessary to clarify the origin of these peculiar PES features, which is beyond the scope of this contribution.

Because both quantities explored and evaluated in this study (i.e., adhesion and potential corrugation) can be correlated to the shear strength of materials,^68^ we hypothesize that MXenes’ interfacial properties can be tailored by manipulating the existing surface terminations. Especially, we theoretically predicted that reducing/limiting −OH groups lead to reduced bilayer adhesion. This fundamentally impacts MXenes’ synthesis and delamination approaches because reduced bilayer adhesion also implies reduced energy for delamination. Moreover, we anticipate that MXenes’ tribological performance can be further optimized by controlling and tailoring the existing surface terminations. We hypothesize that by limiting the percentage of −OH groups, MXenes can provide similar or even better lubricity as other 2D materials such as graphene or MoS_2_.

### 3.4 MXenes Interaction with the Substrate

**Figure 7:**
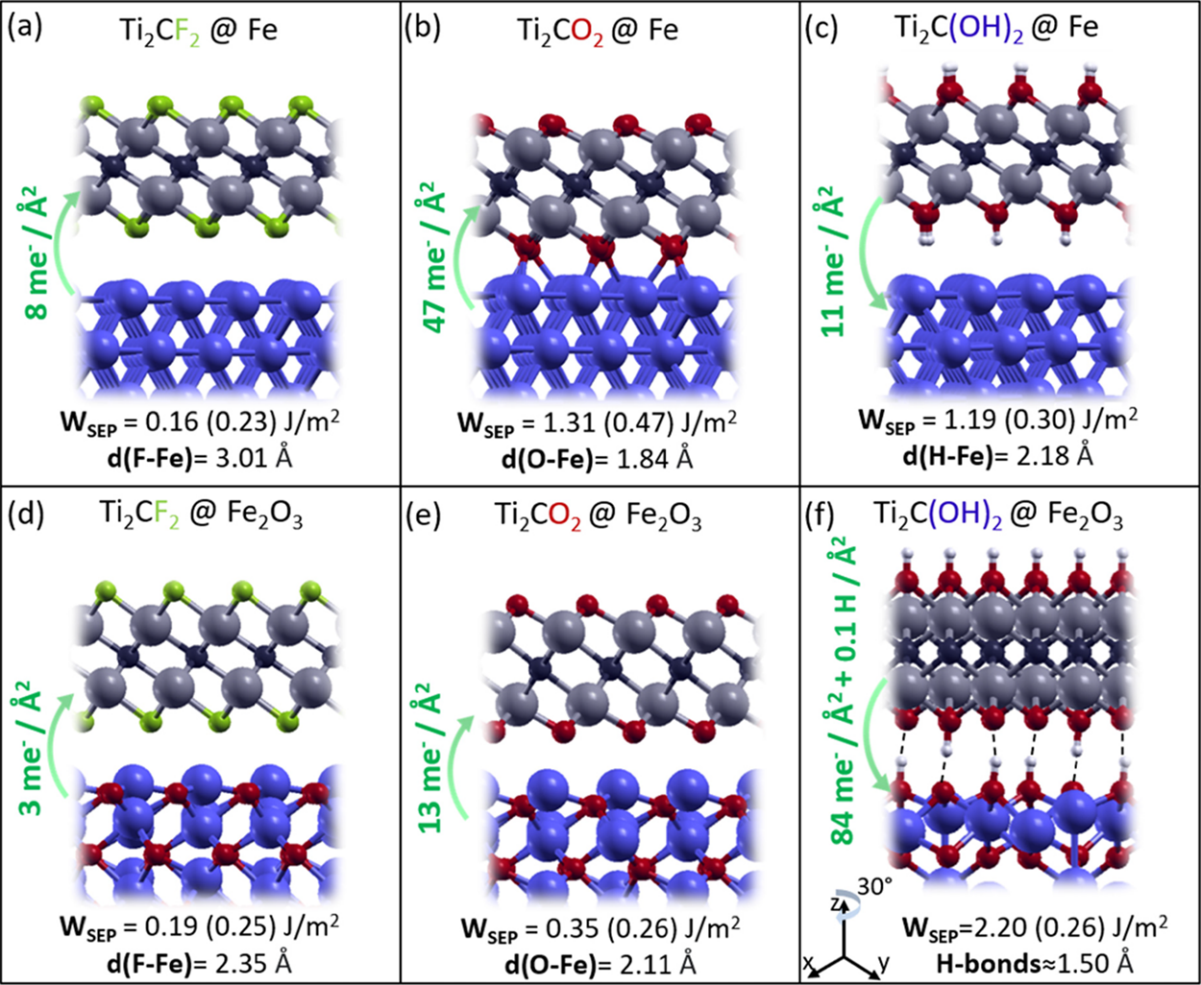
–F, −O, and −OH-terminated MXenes interacting with two steel substrate models: pristine iron (Fe) (a–c) and hematite surfaces (Fe_2_O_3_) (d–f). *W*_SEP_ is reported with its pure dispersive contribution in brackets. The equilibrium distance between the later and surface, *d*, is reported along with the overall charge/matter transfer (green arrows). Atoms are colored as in the previous figures, the Fe atoms being in blue. Dotted lines indicate H-bonds. Please note that the Ti_2_C(OH)_2_@Fe_2_O_3_ view is rotated by 30° around the *z* axes.

In this section, we analyze MXenes’ interaction with ferrous substrates, namely, iron and hematite (Fe_2_O_3_). We considered the effect of homogenous −F, −O, and −OH terminations on both substrates. We also investigated mixed terminations for the iron substrate. However, we did not include mixed terminations on hematite because of the relevant computational effort of simulating Fe_2_O_3_ surfaces. The optimized adsorption configurations are shown in Figure 7, where the adhesion energies are also reported. The dispersive (-*D*) contribution to *W*_SEP_ is explicitly indicated to provide an estimate of the physical forces acting across the interface. The transfer of electronic charge occurring upon layer deposition is also reported. It has been shown that this electronic property correlates very well with interfacial adhesion.^69^ The results indicate that Ti_2_CF_2_ is highly inert and adheres to iron and hematite only via dispersion forces. The long interfacial distance, the minimal charge perturbation occurring with the interface formation, and the predominance of the -*D* component (reported in brackets in Figure 7a) on the *W*_SEP_ support this interpretation. Ti_2_CO_2_ and Ti_2_C(OH)_2_ chemisorb on iron as indicated by the higher value of *W*_SEP_. This outcome arises from different electronic effects occurring across the interface (Figure 7b). Ti_2_CO_2_ partially oxidizes the topmost Fe layer, inducing a relevant charge flow from the substrate to the lubricant. Instead, Ti_2_C(OH)_2_ injects charge into the substrate (Figure 7c), which induces a partial reduction of superficial Fe atoms. Similar effects are observed for the hematite substrates (Figure 7d–f). In this case, Ti_2_C(OH)_2_ transfers both charge and mass (two H atoms per cell) to the substrate, establishing short and strong H-bonds across the interface and leading to a high value of *W*_SEP_. Relevant charge transfer occurs at the interface for interfacial distances below 2 Å, independently from the nature of the interactions. In contrast, for larger distances, the charge transfer between the mated surfaces is hindered.

**Figure 8:**
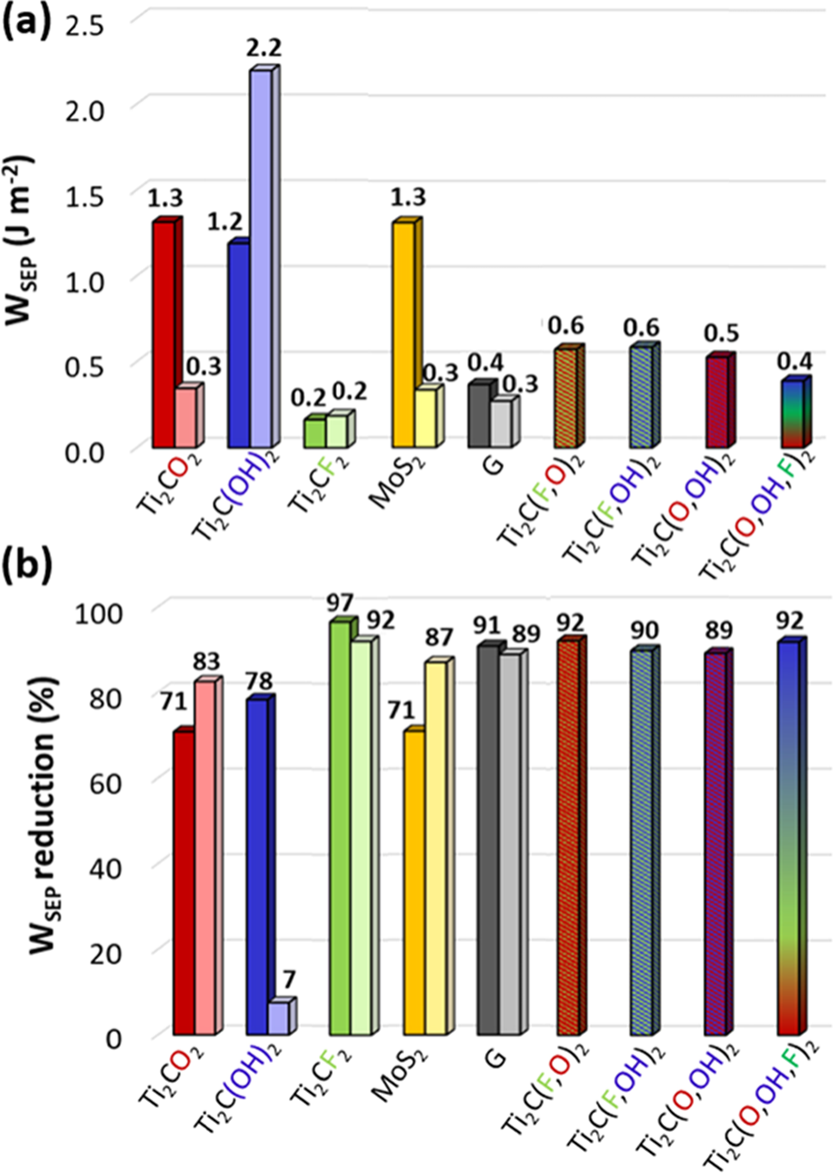
(a) *W*_SEP_ of MXenes with different terminations on Fe (dark color) and Fe_2_O_3_ (pale color). (b) Efficiency of MXenes in reducing the substrate–countersurface adhesion reported as the percentage reduction of *W*_SEP_ with respect to the sealed Fe–Fe and Fe_2_O_3_–Fe_2_O_3_ interfaces. The corresponding values obtained for MoS_2_ and graphene are shown for comparison. Results for mixed termination are reported for Fe only.

An effective solid lubricant should well adhere to the substrate to resist pealing-off during rubbing, but it should also be able to effectively reduce the metal–metal interaction at the micro-asperity contacts. The latter property can be estimated by calculating the reduction of the metal–metal adhesion that is obtained by covering one of the two mating surfaces with a MXenes layer. The results of this analysis are shown in Figure 8, where the MXenes adhesion on the substrates is also reported for comparison.

We observe that Ti_2_C(OH)_2_ MXenes present high adhesion on the substrate (Figure 8a), but it poorly lubricates hematite–hematite contacts (Figure 8b) due to strong H-bond formation across the interface. Ti_2_CF_2_ MXenes demonstrate an outstanding lubricant capability, but weakly bind to both substrates considered (Figure 8a). The adhesion on ferrous surfaces is lower compared to graphene, suggesting a fast removal from the contact zone during rubbing. Interestingly, Ti_2_CO_2_ adheres to the substrate similarly to MoS_2_ and even better than graphene. It also lubricates the considered substrates efficiently, thus representing the best-performing MXene termination among those considered (Figure 8b). The results obtained for the adsorption of MXenes with mixed termination on iron indicate that the simultaneous presence of −O (−OH) and −F atoms reduce the layer adhesion to the substrate (Figure 8a) and enhance the adhesion–reduction capability (Figure 8b). The values of *W*_SEP_ and *W*_SEP_-reduction for the mixed cases are very close to the averages of the corresponding homogenous cases. Intermixing −O and −OH produces, instead, lower adhesion and higher adhesion–reduction than expected, considering the average values obtained for the corresponding homogeneous surfaces.

Finally, we calculated the *W*_SEP_ for two iron surfaces fully covered by MXene layers. The optimized geometries and adhesion values are reported in Figure S10 for different homogenously terminated MXenes. Our results indicate that the presence of the substrate only slightly influences *W*_SEP_. It is considered that, in general, the adhesion correlates well to the PES corrugation.^69^ Therefore, we anticipate that the results discussed in Section 3.3 on the corrugation energy, Δ*W*_SEP_, hold true in the presence of a substrate. This analysis is also supported by the finding reported in a previous study,^70^ where it is demonstrated that the adhesion and shear strength of an iron interface fully covered by graphene are very similar to those obtained for a graphene bilayer.

## 4 Conclusions

In this work, we present a theoretical DFT study aiming at providing an in-depth understanding on the interfacial properties (adhesion) of Ti-based MXenes by considering more realistic models for MXenes’ surface terminations. Initial calculations were devoted to set up a computational scheme that allows for an accurate description of the dispersion forces, avoiding an overestimation of MXenes’ interlayer coupling connected with the use of semi-empirical methods with standard parameters.

Compared to the effects of MXenes’ monolayer thicknesses (*n* = 1 to 3) and their C/N ratio, we demonstrate that surface terminations play the dominant role in determining the interfacial/interlayer properties. For fully −F- and/or −O-terminated MXenes, the interaction between layers is governed by the sum of attractive dispersion forces and electrostatic repulsion between negatively charged surface groups. With predicted values of *W*_SEP_ ≈ 0.16 J m^–2^ and Δ*W*_SEP_ ≈ 0.06 J m^–2^, we demonstrate low interfacial adhesion and, thus, we anticipate an excellent tribological behavior, close to or even better than the best-performing, state-of-the-art 2D materials (e.g., graphene and MoS_2_). In contrast, −OH terminations induce further dipole–dipole interactions between adjacent layers, which are not formed for MXenes terminated by −F and −O. This, in turn, increases the interlayer adhesion and the energy demand to induce interlayer sliding. Interestingly, for MXene bilayers with two or three different terminations covering the surface, the *W*_SEP_ values are not a simple average of the homogeneous cases. Indeed, we found stronger interlayer interactions because of the formation of hydrogen bonds between −OH terminations (H-bond donor) of one layer and −O or −F (H-bond acceptor) of the other layer.

Previous literature results indicate that homogeneous interfaces are more slippery when the MXene termination is −OH than −O.^31^ This result disagrees with our finding, which is based on a methodological approach accurately validated against the higher-level of theory. Our findings have been also verified in a recent experimental work.^34^

The evaluation of the potential energy corrugation Δ*W*_SEP_ under an applied external normal load verified that the load dependence of the resistance to sliding is governed by MXenes’ surface terminations. Ti_2_XT_*x*_ interfaces (with X: C/N, T_*x*_: O and/or F) behave like graphene and MoS_2_ without a notable load dependence of ΔW_SEP_ ranging between 0.06 and 0.12 J m^–2^. The mixed presence of both −OH and −F/–O terminations leads to high potential corrugation that notably increases with load. The highest Δ*W*_SEP_ value is observed for the heterogeneous bilayer Ti_2_CO_2_@Ti_2_C(OH)_2_ (0.48 and 0.56 J/m^2^ at 0 and 10 GPa, respectively). Once again, we highlighted the strong directionality of the hydrogen bond, thus resulting in higher energy barriers.

The surface terminations of MXenes also play a crucial role regarding the interaction with underlying substrates. We studied differently terminated monolayer MXenes on iron and iron oxide to get insights into their ability to lubricate steel. We calculated the layer–substrate adhesion and mated the coated substrate with a countersurface to evaluate the MXenes capability to reduce nano-asperity adhesion. We observe that an increase in the −F concentration weakens layer adhesion to ferrous substrates, which may ease the lubricant removal under sliding conditions. In contrast, −OH terminations anchor the monolayer to the substrate through H-bond and electrostatic interactions but lead to a less efficient lubrication efficiency. MXenes with −O termination adhere well to ferrous surfaces with a lubricant performance similar to graphene and MoS_2_. Considering mixed terminated MXenes, for some compositions such as Ti_2_C(F,O)_2_ and Ti_2_C(F,OH)_2_, both the adhesion to the iron substrate and the reduction of metal–metal adhesion are simply the average of the corresponding homogenous cases. However, our findings reveal that layers with intermixed −OH and −O (i.e., Ti_2_C(OH,O)_2_ and Ti_2_C(O,OH,F)_2_) are more weakly anchored to the substrate and lubricate less the iron–iron contact.

Our computational results indicate that surface terminations are essential for tuning the MXenes tribological properties. By reducing/limiting −OH groups, we demonstrated reduced interlayer binding, which impacts delamination processes as well as the tribological performances. We also observed that by increasing −O terminations, MXenes can better reduce adhesive friction between ferrous micro-asperities, still adhering to the ferrous substrates and thus reducing the lubricant removal during rubbing. Therefore, we hypothesize that MXenes’ (nano)-tribological properties can be further optimized by controlling the surface terminations either by the etching process, for example, by minimizing the F-content in the MXene etchant or by postsynthesis treatments, for example, by reducing the content of −OH terminations by thermal annealing.

## Supporting Information Available

Convergence test for the cutoffs of the wave-function and charge density and the *K*-point grid; high-symmetry lateral positions for MXenes with mixed terminations; optimized geometries of heterogeneous and mixed interfaces; pPESes for all MXene bilayers; computational test at the higher level of theory; and adhesion for MXene layers loaded on the iron substrate (PDF)

## References

[ref1] Naguib M.; Kurtoglu M.; Presser V.; Lu J.; Niu J.; Heon M.; Hultman L.; Gogotsi Y.; Barsoum M. W. Two-Dimensional Nanocrystals Produced by Exfoliation of Ti 3AlC 2. Adv. Mater. 2011, 23, 4248–4253. 10.1002/adma.201102306. 21861270

[ref2] Anasori B.; Lukatskaya M. R.; Gogotsi Y. 2D Metal Carbides and Nitrides (MXenes) for Energy Storage. Nat. Rev. Mater. 2017, 2, 16098 10.1038/natrevmats.2016.98.

[ref3] Pang J.; Mendes R. G.; Bachmatiuk A.; Zhao L.; Ta H. Q.; Gemming T.; Liu H.; Liu Z.; Rummeli M. H. Applications of 2D MXenes in Energy Conversion and Storage Systems. Chem. Soc. Rev. 2019, 48, 72–133. 10.1039/c8cs00324f. 30387794

[ref4] VahidMohammadi A.; Rosen J.; Gogotsi Y. The World of Two-Dimensional Carbides and Nitrides (MXenes). Science 2021, 372, eabf1581 10.1126/science.abf1581. 34112665

[ref5] Kim S. J.; Koh H. J.; Ren C. E.; Kwon O.; Maleski K.; Cho S. Y.; Anasori B.; Kim C. K.; Choi Y. K.; Kim J.; Gogotsi Y.; Jung H. T. Metallic Ti3C2Tx MXene Gas Sensors with Ultrahigh Signal-to-Noise Ratio. ACS Nano 2018, 12, 986–993. 10.1021/acsnano.7b07460. 29368519

[ref6] Yu T.; Breslin C. B. Review—Two-Dimensional Titanium Carbide MXenes and Their Emerging Applications as Electrochemical Sensors. J. Electrochem. Soc. 2020, 167, 037514 10.1149/2.0142003JES.

[ref7] Yun T.; Kim H.; Iqbal A.; Cho Y. S.; Lee G. S.; Kim M. K.; Kim S. J.; Kim D.; Gogotsi Y.; Kim S. O.; Koo C. M. Electromagnetic Shielding of Monolayer MXene Assemblies. Adv. Mater. 2020, 32, 1906769 10.1002/adma.201906769. 31971302

[ref8] Han M.; Shuck C. E.; Rakhmanov R.; Parchment D.; Anasori B.; Koo C. M.; Friedman G.; Gogotsi Y. Beyond Ti3C2Tx: MXenes for Electromagnetic Interference Shielding. ACS Nano 2020, 14, 5008–5016. 10.1021/acsnano.0c01312. 32163265

[ref9] Sharma N.; Ojha H.; Bharadwaj A.; Pathak D. P.; Sharma R. K. Preparation and Catalytic Applications of Nanomaterials: A Review. RSC Adv. 2015, 5, 53381–53403. 10.1039/C5RA06778B.

[ref10] Liu A.; Liang X.; Ren X.; Guan W.; Gao M.; Yang Y.; Yang Q.; Gao L.; Li Y.; Ma T. Recent Progress in MXene-Based Materials: Potential High-Performance Electrocatalysts. Adv. Funct. Mater. 2020, 30, 2003437 10.1002/adfm.202003437.

[ref11] Morales-Garciá Á.; Calle-Vallejo F.; Illas F. MXenes: New Horizons in Catalysis. ACS Catal. 2020, 10, 13487–13503. 10.1021/acscatal.0c03106.

[ref12] Wyatt B. C.; Rosenkranz A.; Anasori B. 2D MXenes: Tunable Mechanical and Tribological Properties. Adv. Mater. 2021, 33, 2007973 10.1002/adma.202007973. 33738850

[ref13] Grützmacher P. G.; Suarez S.; Tolosa A.; Gachot C.; Song G.; Wang B.; Presser V.; Mücklich F.; Anasori B.; Rosenkranz A. Superior Wear-Resistance of Ti3C2TxMultilayer Coatings. ACS Nano 2021, 15, 8216–8224. 10.1021/acsnano.1c01555. 33822595

[ref14] Marian M.; Feile K.; Rothammer B.; Bartz M.; Wartzack S.; Seynstahl A.; Tremmel S.; Krauß S.; Merle B.; Böhm T.; Wang B.; Wyatt B. C.; Anasori B.; Rosenkranz A. Ti3C2Tx Solid Lubricant Coatings in Rolling Bearings with Remarkable Performance beyond State-of-the-Art Materials. Appl. Mater. Today 2021, 25, 101202 10.1016/j.apmt.2021.101202.

[ref15] Alhabeb M.; Maleski K.; Anasori B.; Lelyukh P.; Clark L.; Sin S.; Gogotsi Y. Guidelines for Synthesis and Processing of Two-Dimensional Titanium Carbide (Ti3C2Tx MXene). Chem. Mater. 2017, 29, 7633–7644. 10.1021/acs.chemmater.7b02847.

[ref16] Deysher G.; Shuck C. E.; Hantanasirisakul K.; Frey N. C.; Foucher A. C.; Maleski K.; Sarycheva A.; Shenoy V. B.; Stach E. A.; Anasori B.; Gogotsi Y. Synthesis of Mo4VAlC4 MAX Phase and Two-Dimensional Mo4VC4 MXene with Five Atomic Layers of Transition Metals. ACS Nano 2020, 14, 204–217. 10.1021/acsnano.9b07708. 31804797

[ref17] Naguib M.; Gogotsi Y. Synthesis of Two-Dimensional Materials by Selective Extraction. Acc. Chem. Res. 2015, 48, 128–135. 10.1021/ar500346b. 25489991

[ref18] Xiao X.; Wang H.; Urbankowski P.; Gogotsi Y. Topochemical Synthesis of 2D Materials. Chem. Soc. Rev. 2018, 47, 8744–8765. 10.1039/c8cs00649k. 30302443

[ref19] Hope M. A.; Forse A. C.; Griffith K. J.; Lukatskaya M. R.; Ghidiu M.; Gogotsi Y.; Grey C. P. NMR Reveals the Surface Functionalisation of Ti3C2 MXene. Phys. Chem. Chem. Phys. 2016, 18, 5099–5102. 10.1039/c6cp00330c. 26818187

[ref20] Harris K. J.; Bugnet M.; Naguib M.; Barsoum M. W.; Goward G. R. Direct Measurement of Surface Termination Groups and Their Connectivity in the 2D MXene V2CTx Using NMR Spectroscopy. J. Phys. Chem. C 2015, 119, 13713–13720. 10.1021/acs.jpcc.5b03038.

[ref21] Persson I.; Näslund L. Å.; Halim J.; Barsoum M. W.; Darakchieva V.; Palisaitis J.; Rosen J.; Persson P. O. Å. On the Organization and Thermal Behavior of Functional Groups on Ti3C2 MXene Surfaces in Vacuum. 2D Mater 2018, 5, 015002 10.1088/2053-1583/aa89cd.

[ref22] Seredych M.; Shuck C. E.; Pinto D.; Alhabeb M.; Precetti E.; Deysher G.; Anasori B.; Kurra N.; Gogotsi Y. High-Temperature Behavior and Surface Chemistry of Carbide MXenes Studied by Thermal Analysis. Chem. Mater. 2019, 31, 3324–3332. 10.1021/acs.chemmater.9b00397.

[ref23] Zhang S.; Ma T.; Erdemir A.; Li Q. Tribology of Two-Dimensional Materials: From Mechanisms to Modulating Strategies. Mater. Today 2019, 26, 67–86. 10.1016/j.mattod.2018.12.002.

[ref24] Berman D.; Erdemir A.; Sumant A. V. Approaches for Achieving Superlubricity in Two-Dimensional Materials. ACS Nano 2018, 12, 2122–2137. 10.1021/acsnano.7b09046. 29522673

[ref25] Rosenkranz A.; Grützmacher P. G.; Espinoza R.; Fuenzalida V. M.; Blanco E.; Escalona N.; Gracia F. J.; Villarroel R.; Guo L.; Kang R.; Mücklich F.; Suarez S.; Zhang Z. Multi-Layer Ti3C2Tx-Nanoparticles (MXenes) as Solid Lubricants – Role of Surface Terminations and Intercalated Water. Appl. Surf. Sci. 2019, 494, 13–21. 10.1016/j.apsusc.2019.07.171.

[ref26] Rodriguez A.; Jaman M. S.; Acikgoz O.; Wang B.; Yu J.; Grützmacher P. G.; Rosenkranz A.; Baykara M. Z. The Potential of Ti3C2TX Nano-Sheets (MXenes) for Nanoscale Solid Lubrication Revealed by Friction Force Microscopy. Appl. Surf. Sci. 2021, 535, 147664 10.1016/j.apsusc.2020.147664.

[ref27] Marian M.; Feile K.; Rothammer B.; Bartz M.; Wartzack S.; Seynstahl A.; Tremmel S.; Krauß S.; Merle B.; Böhm T.; Wang B.; Wyatt B. C.; Anasori B.; Rosenkranz A. Ti3C2T Solid Lubricant Coatings in Rolling Bearings with Remarkable Performance beyond State-of-the-Art Materials. Appl. Mater. Today 2021, 25, 101202 10.1016/j.apmt.2021.101202.

[ref28] Huang S.; Mutyala K. C.; Sumant A. V.; Mochalin V. N. Achieving Superlubricity with 2D Transition Metal Carbides (MXenes) and MXene/Graphene Coatings. Mater. Today Adv. 2021, 9, 100133 10.1016/j.mtadv.2021.100133.

[ref29] Marian M.; Song G. C.; Wang B.; Fuenzalida V. M.; Krauß S.; Merle B.; Tremmel S.; Wartzack S.; Yu J.; Rosenkranz A. Effective Usage of 2D MXene Nanosheets as Solid Lubricant – Influence of Contact Pressure and Relative Humidity. Appl. Surf. Sci. 2020, 531, 147311 10.1016/j.apsusc.2020.147311.

[ref30] Hu T.; Hu M.; Li Z.; Zhang H.; Zhang C.; Wang J.; Wang X. Interlayer Coupling in Two-Dimensional Titanium Carbide MXenes. Phys. Chem. Chem. Phys. 2016, 18, 20256–20260. 10.1039/c6cp01699e. 27212073

[ref31] Zhang D.; Ashton M.; Ostadhossein A.; Van Duin A. C. T.; Hennig R. G.; Sinnott S. B. Computational Study of Low Interlayer Friction in Tin+1Cn (n = 1, 2, and 3) MXene. ACS Appl. Mater. Interfaces 2017, 9, 34467–34479. 10.1021/acsami.7b09895. 28884568

[ref32] Zhang H.; Fu Z. H.; Legut D.; Germann T. C.; Zhang R. F. Stacking Stability and Sliding Mechanism in Weakly Bonded 2D Transition Metal Carbides by van Der Waals Force. RSC Adv. 2017, 7, 55912–55919. 10.1039/C7RA11139H.

[ref33] Zhang Y.; Chen X.; Arramel; Augustine K. B.; Zhang P.; Jiang J.; Wu Q.; Li N. Atomic-Scale Superlubricity in Ti2CO2@MoS2Layered Heterojunctions Interface: A First Principles Calculation Study. ACS Omega 2021, 6, 9013–9019. 10.1021/acsomega.1c00036. 33842771 PMC8028160

[ref34] Serles P.; Hamidinejad M.; Demingos P. G.; Ma L.; Barri N.; Taylor H.; Singh C. V.; Park C. B.; Filleter T. Friction of Ti3C2Tx MXenes. Nano Lett. 2022, 22, 3356–3363. 10.1021/acs.nanolett.2c00614. 35385668

[ref35] Reguzzoni M.; Fasolino A.; Molinari E.; Righi M. C. Potential Energy Surface for Graphene on Graphene: Ab Initio Derivation, Analytical Description, and Microscopic Interpretation. Phys. Rev. B Condens. Matter Mater. Phys. 2012, 86, 245434 10.1103/PhysRevB.86.245434.

[ref36] Levita G.; Cavaleiro A.; Molinari E.; Polcar T.; Righi M. C. Sliding Properties of MoS2 Layers: Load and Interlayer Orientation Effects. J. Phys. Chem. C 2014, 118, 13809–13816. 10.1021/jp4098099.

[ref37] Levita G.; Molinari E.; Polcar T.; Righi M. C. First-Principles Comparative Study on the Interlayer Adhesion and Shear Strength of Transition-Metal Dichalcogenides and Graphene. Phys. Rev. B Condens. Matte Mater. Phys. 2015, 92, 085434 10.1103/PhysRevB.92.085434.

[ref38] Losi G.; Cutini M.; Restuccia P.; Righi M. C. Modeling Phosphorene and MoS2 Interacting with Iron: Lubricating Effects Compared to Graphene. J. Nanostruct. Chem. 2022, 1–9. 10.1007/s40097-022-00478-1.

[ref39] Giannozzi P.; Baroni S.; Bonini N.; Calandra M.; Car R.; Cavazzoni C.; Ceresoli D.; Chiarotti G. L.; Cococcioni M.; Dabo I.; Dal Corso A.; De Gironcoli S.; Fabris S.; Fratesi G.; Gebauer R.; Gerstmann U.; Gougoussis C.; Kokalj A.; Lazzeri M.; Martin-Samos L.; Marzari N.; Mauri F.; Mazzarello R.; Paolini S.; Pasquarello A.; Paulatto L.; Sbraccia C.; Scandolo S.; Sclauzero G.; Seitsonen A. P.; Smogunov A.; Umari P.; Wentzcovitch R. M. QUANTUM ESPRESSO: A Modular and Open-Source Software Project for Quantum Simulations of Materials. J. Phys. Condens. Matter 2009, 21, 395502 10.1088/0953-8984/21/39/395502. 21832390

[ref40] Perdew J. P.; Burke K.; Ernzerhof M. Generalized Gradient Approximation Made Simple. Phys. Rev. Lett. 1996, 77, 3865–3868. 10.1103/PhysRevLett.77.3865. 10062328

[ref41] Monkhorst H. J.; Pack J. D. Special Points for Brillouin-Zone Integrations. Phys. Rev. B 1976, 13, 5188–5192. 10.1103/PhysRevB.13.5188.

[ref42] Anisimov V. I.; Zaanen J.; Andersen O. K. Band Theory and Mott Insulators: Hubbard U Instead of Stoner I. Phys. Rev. B 1991, 44, 943–954. 10.1103/PhysRevB.44.943. 9999600

[ref43] Wang R. B.; Hellman A. Initial Water Adsorption on Hematite (α -Fe2O3) (0001): A DFT + U Study. J. Chem. Phys. 2018, 148, 094705 10.1063/1.5020358.

[ref44] Nguyen M. T.; Seriani N.; Gebauer R. Water Adsorption and Dissociation on α-Fe 2 O 3 (0001): PBE+U Calculations. J. Chem. Phys. 2013, 138, 194709 10.1063/1.4804999. 23697432

[ref45] Dzade N. Y.; Roldan A.; de Leeuw N. H. A Density Functional Theory Study of the Adsorption of Benzene on Hematite (α-Fe2O3) Surfaces. Minerals 2014, 4, 89–115. 10.3390/min4010089.

[ref46] Stirner T.; Scholz D.; Sun J. Convergence of Surface Energy Calculations for Various Methods: (0 0 1), (0 1 2), (1 0 0) Hematite and the Applicability of the Standard Approach. J. Phys. Condens. Matter 2020, 32, 185002 10.1088/1361-648X/ab6f88. 31978904

[ref47] Spencer M. J. S.; Hung A.; Snook I. K.; Yarovsky I. Density Functional Theory Study of the Relaxation and Energy of Iron Surfaces. Surf. Sci. 2002, 513, 389–398. 10.1016/S0039-6028(02)01809-5.

[ref48] Grimme S. Semiempirical GGA-Type Density Functional Constructed with a Long-Range Dispersion Correction. J. Comput. Chem. 2006, 27, 1787–1799. 10.1002/jcc.20495. 16955487

[ref49] Grimme S.; Antony J.; Ehrlich S.; Krieg H. A Consistent and Accurate Ab Initio Parametrization of Density Functional Dispersion Correction (DFT-D) for the 94 Elements H-Pu. J. Chem. Phys. 2010, 132, 154104 10.1063/1.3382344. 20423165

[ref50] Tkatchenko A.; Scheffler M. Accurate Molecular van Der Waals Interactions from Ground-State Electron Density and Free-Atom Reference Data. Phys. Rev. Lett. 2009, 102, 073005 10.1103/PhysRevLett.102.073005. 19257665

[ref51] Tkatchenko A.; Distasio R. A.; Car R.; Scheffler M. Accurate and Efficient Method for Many-Body van Der Waals Interactions. Phys. Rev. Lett. 2012, 108, 236402 10.1103/PhysRevLett.108.236402. 23003978

[ref52] Steinmann S. N.; Corminboeuf C. A Generalized-Gradient Approximation Exchange Hole Model for Dispersion Coefficients. J. Chem. Phys. 2011, 134, 044117 10.1063/1.3545985. 21280697

[ref53] Lee K.; Murray É. D.; Kong L.; Lundqvist B. I.; Langreth D. C. Higher-Accuracy van Der Waals Density Functional. Phys. Rev. B 2010, 82, 081101 10.1103/PhysRevB.82.081101.

[ref54] Sun J.; Ruzsinszky A.; Perdew J. Strongly Constrained and Appropriately Normed Semilocal Density Functional. Phys. Rev. Lett. 2015, 115, 036402 10.1103/PhysRevLett.115.036402. 26230809

[ref55] Kaltak M.; Klimeš J.; Kresse G. Cubic Scaling Algorithm for the Random Phase Approximation: Self-Interstitials and Vacancies in Si. Phys. Rev. B Condens. Matter Mater. Phys. 2014, 90, 054115 10.1103/PhysRevB.90.054115.

[ref56] Usvyat D.; Maschio L.; Schütz M. Periodic Local MP2 Method Employing Orbital Specific Virtuals. J. Chem. Phys. 2015, 143, 102805 10.1063/1.4921301. 26373998

[ref57] Cutini M.; Maschio L.; Ugliengo P. Exfoliation Energy of Layered Materials by DFT-D: Beware of Dispersion!. J. Chem. Theory Comput. 2020, 16, 5244–5252. 10.1021/acs.jctc.0c00149. 32609519 PMC8009511

[ref58] Kresse G.; Jürgen F. Efficient Iterative Schemes for Ab Initiototal-Energy Calculations Using a Plane-Wave Basis Set. Phys. Rev. B 1996, 54, 11169 10.1103/PhysRevB.54.11169. 9984901

[ref59] Henkelman G.; Arnaldsson A.; Jónsson H. A Fast and Robust Algorithm for Bader Decomposition of Charge Density. Comput. Mater. Sci. 2006, 36, 354–360. 10.1016/j.commatsci.2005.04.010.

[ref60] Grimme S.; Hansen A.; Brandenburg J. G.; Bannwarth C. Dispersion-Corrected Mean-Field Electronic Structure Methods. Chem. Rev. 2016, 116, 5105–5154. 10.1021/acs.chemrev.5b00533. 27077966

[ref61] Cutini M.; Bocus M.; Ugliengo P. Decoding Collagen Triple Helix Stability by Means of Hybrid DFT Simulations. J. Phys. Chem. B 2019, 123, 7354–7364. 10.1021/acs.jpcb.9b05222. 31365821

[ref62] Cutini M.; Pantaleone S.; Ugliengo P. Elucidating the Nature of Interactions in Collagen Triple-Helix Wrapping. J. Phys. Chem. Lett. 2019, 10, 7644–7649. 10.1021/acs.jpclett.9b03125. 31738560

[ref63] Cutini M.; Bechis I.; Corno M.; Ugliengo P. Balancing Cost and Accuracy in Quantum Mechanical Simulations on Collagen Protein Models. J. Chem. Theory Comput. 2021, 17, 2566–2574. 10.1021/acs.jctc.1c00015. 33754704

[ref64] Cutini M.; Civalleri B.; Corno M.; Orlando R.; Brandeburg J. G.; Maschio L.; Ugliengo P. Assessment of Different Quantum Mechanical Methods for the Prediction of Structure and Cohesive Energy of Molecular Crystals. J. Chem. Theory Comput. 2016, 12, 3340–3352. 10.1021/acs.jctc.6b00304. 27304925

[ref65] Caldeweyher E.; Mewes J. M.; Ehlert S.; Grimme S. Extension and Evaluation of the D4 London-Dispersion Model for Periodic Systems. Phys. Chem. Chem. Phys. 2020, 22, 8499–8512. 10.1039/D0CP00502A. 32292979

[ref66] Li Y.; Huang S.; Wei C.; Zhou D.; Li B.; Wu C.; Mochalin V. N. Adhesion between MXenes and Other 2D Materials. ACS Appl. Mater. Interfaces 2021, 13, 4682–4691. 10.1021/acsami.0c18624. 33433988

[ref67] Sang X.; Xie Y.; Lin M. W.; Alhabeb M.; Van Aken K. L.; Gogotsi Y.; Kent P. R. C.; Xiao K.; Unocic R. R. Atomic Defects in Monolayer Titanium Carbide (Ti3C2Tx) MXene. ACS Nano 2016, 10, 9193–9200. 10.1021/acsnano.6b05240. 27598326

[ref68] Wolloch M.; Losi G.; Ferrario M.; Righi M. C. High-Throughput Screening of the Static Friction and Ideal Cleavage Strength of Solid Interfaces. Sci. Rep. 2019, 9, 17062 10.1038/s41598-019-49907-2. 31745097 PMC6863866

[ref69] Wolloch M.; Levita G.; Restuccia P.; Righi M. C. Interfacial Charge Density and Its Connection to Adhesion and Frictional Forces. Phys. Rev. Lett. 2018, 121, 26804 10.1103/PhysRevLett.121.026804. 30085711

[ref70] Restuccia P.; Righi M. C. Tribochemistry of Graphene on Iron and Its Possible Role in Lubrication of Steel. Carbon N. Y. 2016, 106, 118–124. 10.1016/j.carbon.2016.05.025.

