# Supplementary material for: Nanoscale MXene Interlayer and Substrate Adhesion for Lubrication: A Density Functional Theory Study
Source: ACS Appl Nano Mater. 2022 Aug 8;5(8):10516–27. doi: 10.1021/acsanm.2c01847 (PMC9425433; doi:10.1021/acsanm.2c01847)
Supplement: Supplementary file 1 — an2c01847_si_001.pdf [file an2c01847_si_001.pdf]

Supporting Information

Nanoscale MXene Interlayer and Substrate Adhesion for Lubrication: A Density Functional Theory Study

Edoardo Marquis, Michele Cutini, Babak Anasori, Andreas Rosenkranz, and Maria Clelia Righi*

E. Marquis, M. Cutini, Prof. M. C. Righi

Department of Physics and Astronomy, Alma Mater Studiorum – University of Bologna, Viale Berti Pichat 6/2, 40127, Bologna, Italy

E-mail: clelia.righi@unibo.it

Prof. B. Anasori

Department of Mechanical and Energy Engineering, and Integrated Nanosystems Development Institute, Indiana University-Purdue University Indianapolis, Indianapolis, IN 46202, US

Prof. A. Rosenkranz

Department of Chemical Engineering, Biotechnology and Materials, University of Chile, Avenida Beaucheff 851, 8370456, Santiago de Chile, Chile

Computational Details

MXenes are modeled with hexagonal cells. Here we describe the procedure employed for the evaluation of the ‘a’ lattice parameter of equilibrium. We fixed ‘c’ in order to ensure a vacuum region of about 15 Å along z, then we performed multiple relaxations of atomic coordinates while fixing the ‘a’ parameter of the cell at different values. From the data pairs (‘a’ parameter; Energy), we used the ordinary least squares regression to fit a parabolic function thus identifying the minimum. In Figure S1 we reported the convergence test for the cutoffs of the wave-function and charge density and the K-points grid.

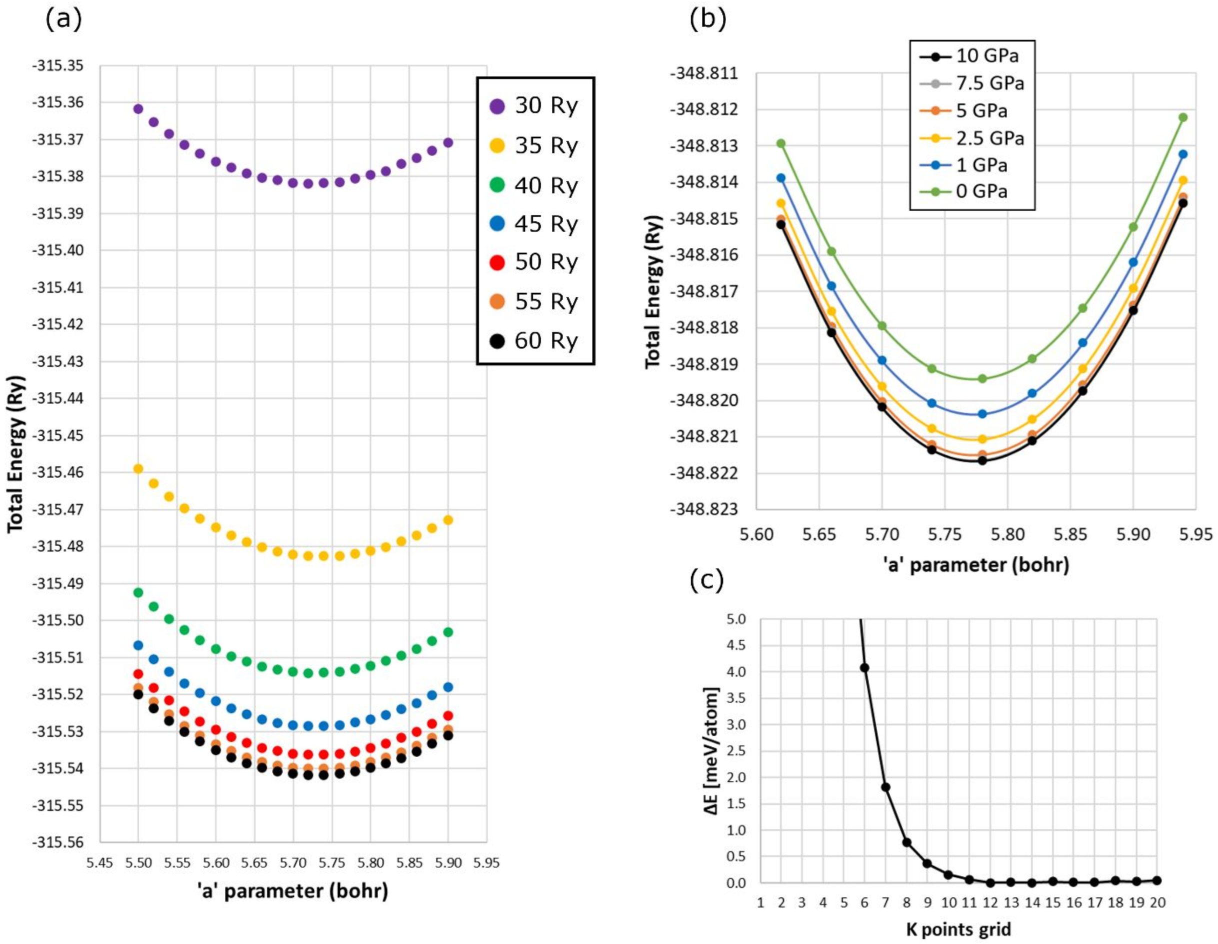

Figure S1. (a) convergence test for the electronic wave-function cutoffs: the optimization of the ‘a’ lattice parameter is repeated at different cutoff values (the charge density cutoff is always set as 8 times the wave-function cutoff). (b) the procedure to find the equilibrium ‘a’ value is repeated in the presence of a perpendicular load: the equilibrium value is not affected by the load entity. (c) convergence test for the NxNx1 Monkhorst-Pack grid; the convergence is reached with a grid of 12x12x1.

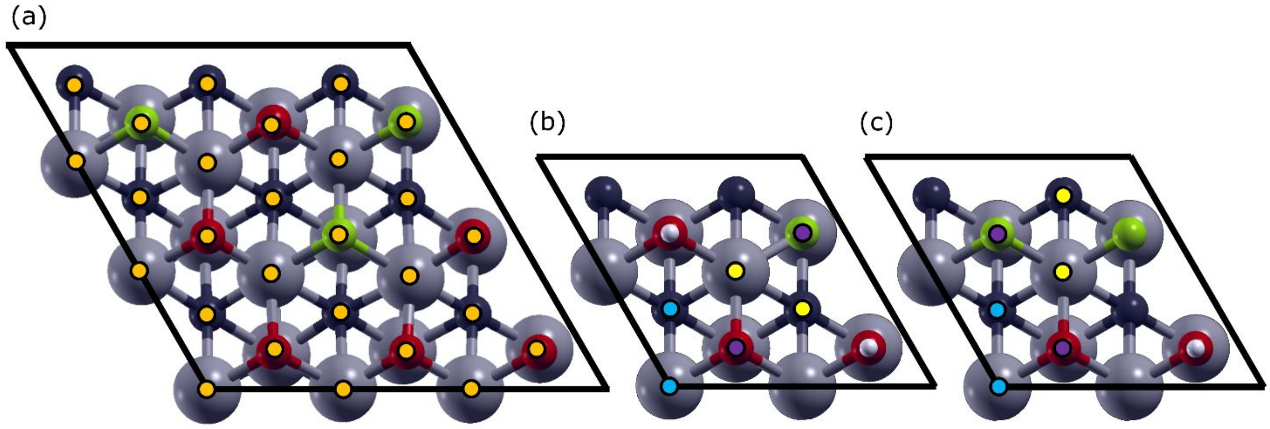

Figure S2. High-symmetry lateral positions tested for the calculation of adhesion energy of MXenes with mixed terminations (T_x). (a) exemplarily represent a fully mixed surface. The 27 lateral positions considered for the identification of the most stable stacking are highlighted with orange circles. (b) is taken as an example for MXenes containing two different terminations in the ratio 3:1 or 1:3, while (c) is for 2:2 ratios. For the cases b and c, the upper MXene is placed above the 6 non-equivalent atoms highlighted with circles: the two terminations (in purple), the Ti and C atoms closest to a specific T_x (-F in the reported example, in yellow), and a Ti and C closer to the other T_x (-OH in the example, in blue).

The calculations of section 3.1 (Figure 2), concerning the analysis of dispersion force's role, were carried out using the Vienna *Ab initio* Simulation Package (VASP) code. The work of separation values with different dispersion schemes was evaluated by performing single-point energy calculations on the structures optimized with other methods. For the Mg and Ca hydroxides, the geometries have been relaxed with B3LYP-D_{NG}, employing a TZP basis set (see "https://doi.org/10.1021/acs.jctc.0c00149" for more details). For MXenes the structures were optimized with PBE-D_{NG} method under the plane-wave pseudopotential approach, which is also the one selected and employed in our work.

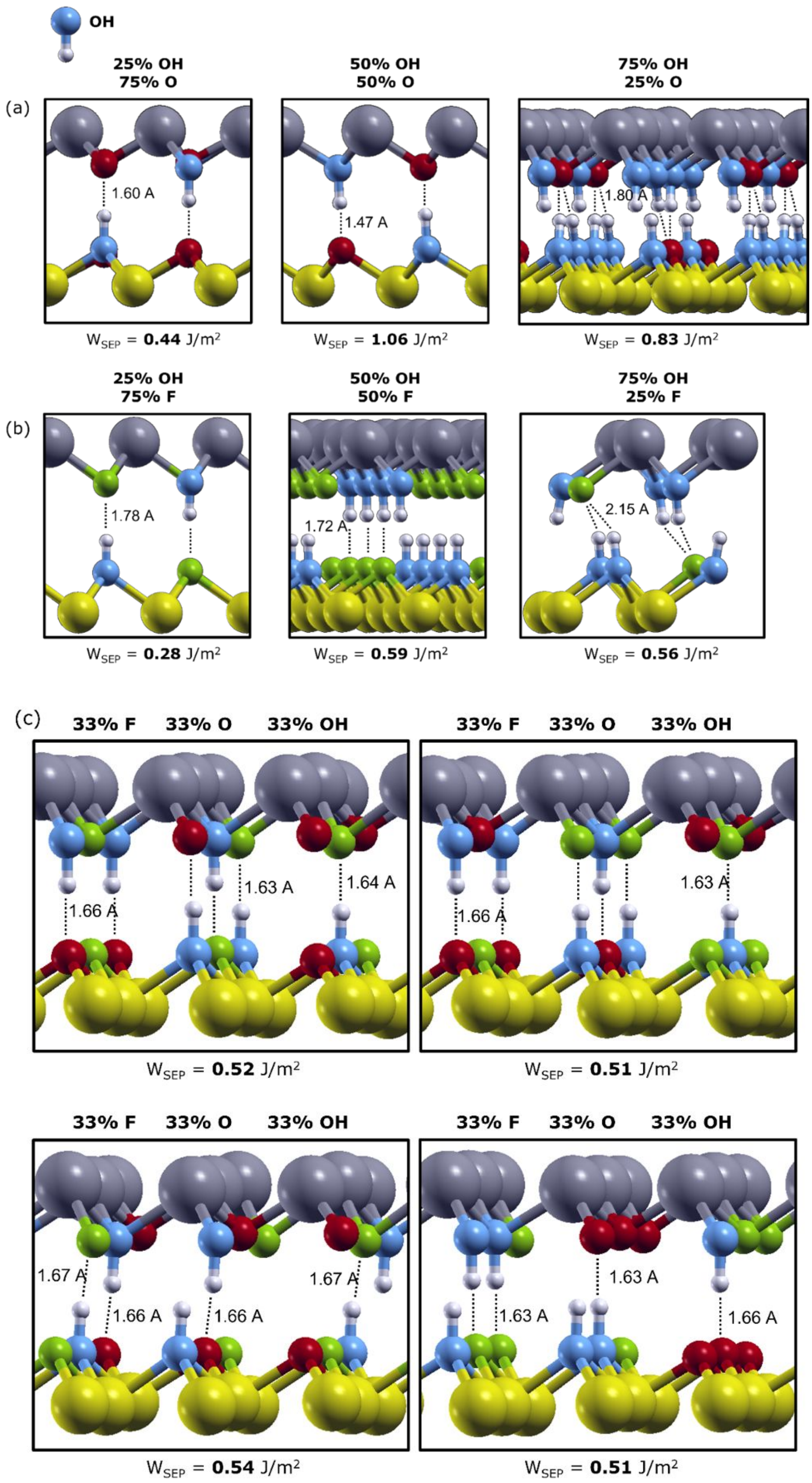

Figure S3. (a-b) Optimized geometries for bilayers with two different T_x containing -OH and (c) optimized geometries for bilayers with three different T_x. For a better visualisation, the oxygen of hydroxyls is colored in light blue, and the Ti atoms of the bottom layer closest to the interface are in yellow. The inner atoms of the structures have been omitted for clarity. Hydrogen bond distances are indicated with dashed lines.

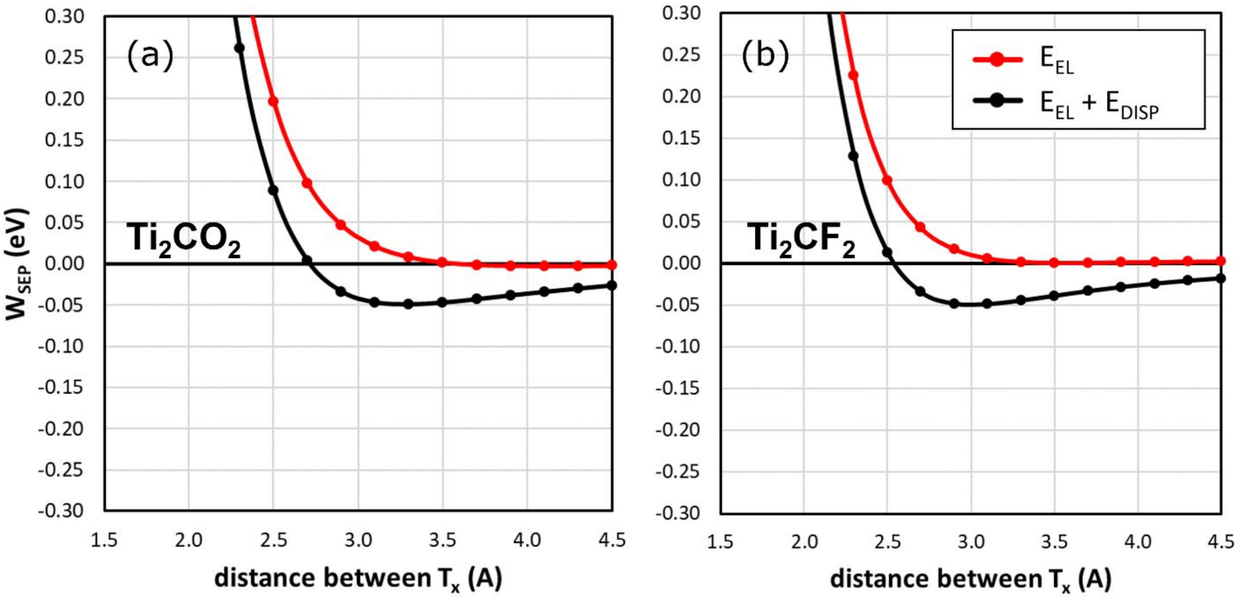

Figure S4. W_{SEP} values as a function of distance between (a) -F for two paired Ti_2CF_2 and (b) -O for two paired Ti_2CO_2. The red line describes the interaction energy when the D_{NG} dispersion correction is "turned off". Without the presence of the dispersion correction the two coupled layers would move to infinite distance due to the electrostatic repulsion between negatively charged terminations.

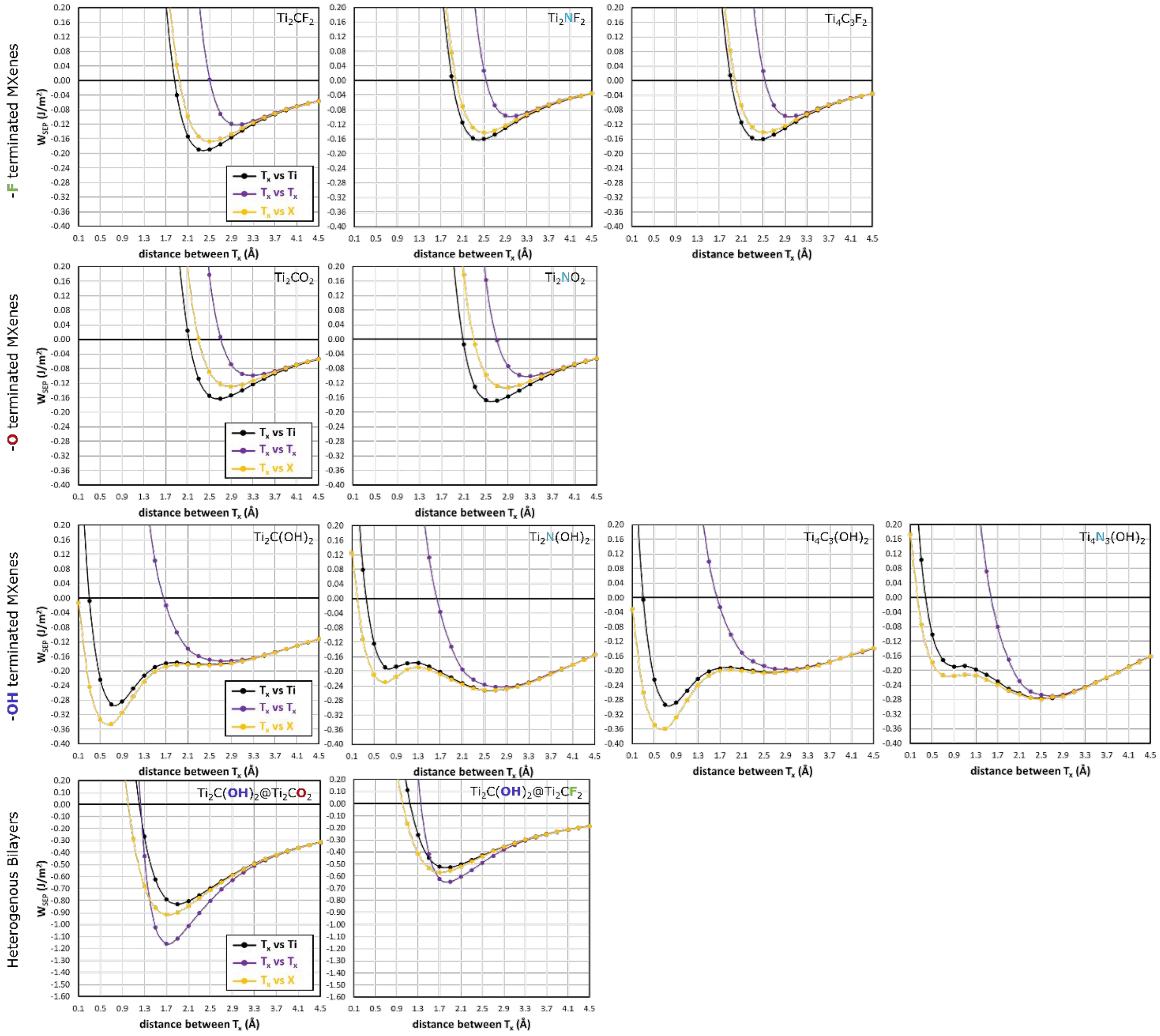

Figure S5. Perpendicular PES (pPES) for the paired MXenes. The work of separation W_{SEP} is shown as a function of the distance between –F, –O, or –H. The black line refers to the configuration where the termination of the top layer faces the bottom layer pointing towards the metal (T_x versus Ti), the yellow line is when it faces the carbon/nitrogen (T_x versus C/N), and the purple one when the terminations are pointing towards each other (T_x versus T_x).

The unexpected ΔW_{SEP} difference at zero-load between $Ti_2C(OH)_2$ and $Ti_2N(OH)_2$ bilayers, can be explained by comparing their pPESes with respect to different lateral positions. **Figure S6** shows pPESes for the two lateral positions corresponding to the minimum of W_{SEP} (yellow line, when the –OH points towards the C/N) and the maximum of W_{SEP} (blue line, when the –OHs point towards each other), respectively. The difference in the energy between the yellow and the blue lines relates to the corrugation ΔW_{SEP}. It can be observed that both pPESes for $Ti_2C(OH)_2$ and $Ti_2N(OH)_2$ present two distinct minima. In the case of the C-containing MXene, the deepest minimum is separated by a 0.23 J m^{-2} from the maximum (Figure S6a), while the separation is largely reduced for the N-containing MXenes ($Ti_2N(OH)_2$), thus leading to a very small corrugation. However, that ΔW_{SEP} difference does not persist in the presence of an applied load as shown in Figure 5b in the main text. This can be explained once again by taking Figure S6b into account. Under the effect of an external load, the left minimum of the yellow line becomes available for $Ti_2N(OH)_2$ thus leading to a similar ΔW_{SEP} compared to the $Ti_2C(OH)_2$ bilayer.

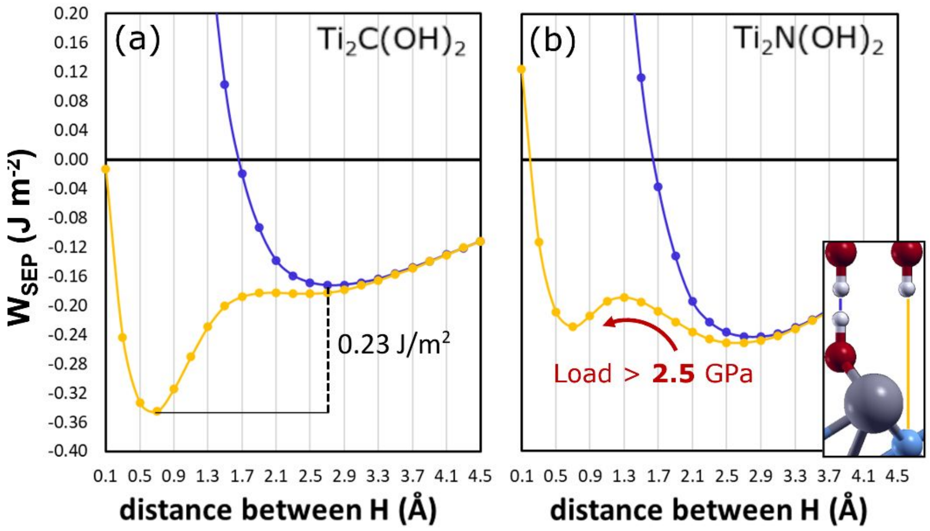

Figure S6. Perpendicular potential energy surface (pPES) for a) $Ti_2C(OH)_2$ and b) $Ti_2N(OH)_2$. For both systems, the blue line refers to the lateral position, for which hydroxyls point towards each other, while the yellow line represents the situation for which the -OH is point towards carbon (left) or nitrogen (right), respectively.

Since the pPES of the homogeneous bilayer $Ti_2C(OH)_2$@$Ti_2C(OH)_2$ calculated with PBE-D_{NG} was not conventional, as it predicts two distinct minima, we repeated the calculations with the SCAN functional. The SCAN energy estimations are run on the PBE-DNG relaxed geometries. In Figure S6 the two pPES are compared.

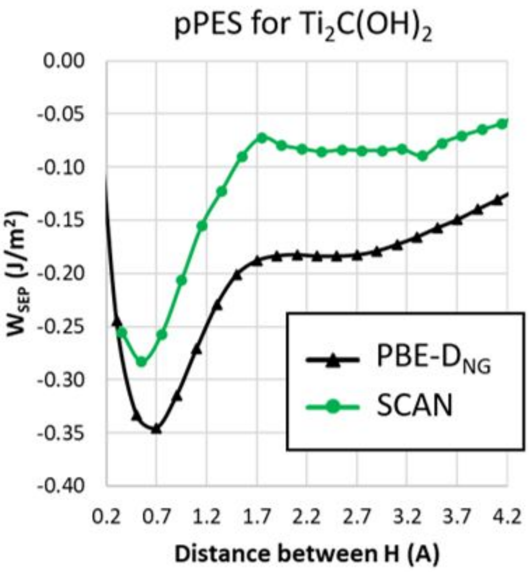

Figure S7. Perpendicular PES of the homogeneous bilayer $Ti_2C(OH)_2$@$Ti_2C(OH)_2$ calculated with PBE-D_{NG} (black line) and with SCAN (green line). The energetic profiles provided by the two methods are similar, apart from the upward shift of the green line. The SCAN energy estimations are run on the PBE-D_{NG} relaxed geometries.

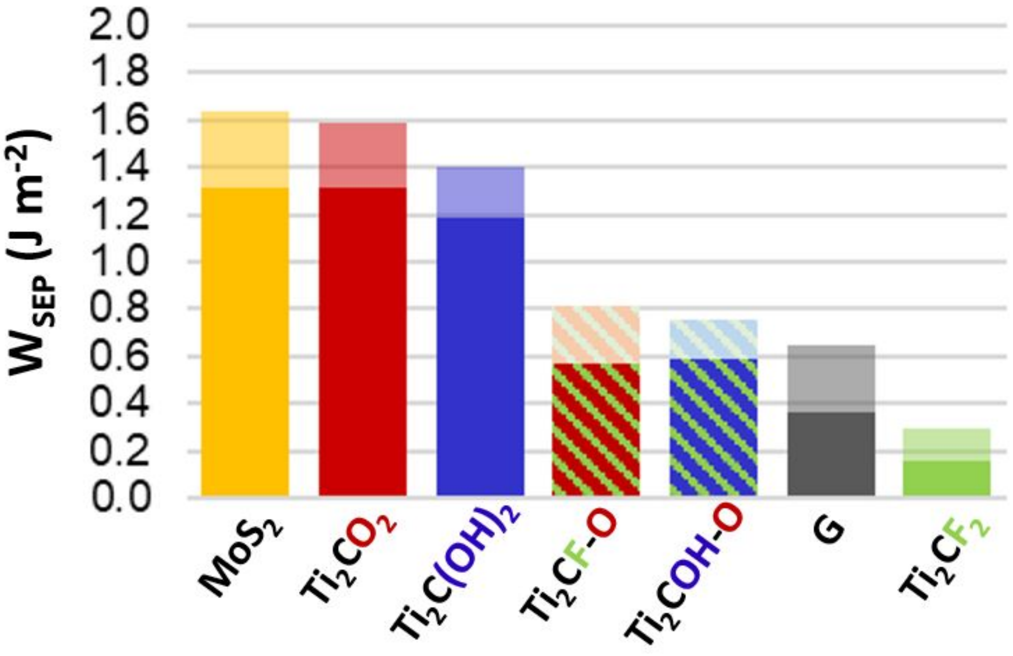

Figure S8. W_{SEP} for different MXenes on (110) Fe surface as well as MoS_2 and graphene. Shaded/full bars indicate the values computed using standard-D2/D_{NG} parameters for the Fe atom.

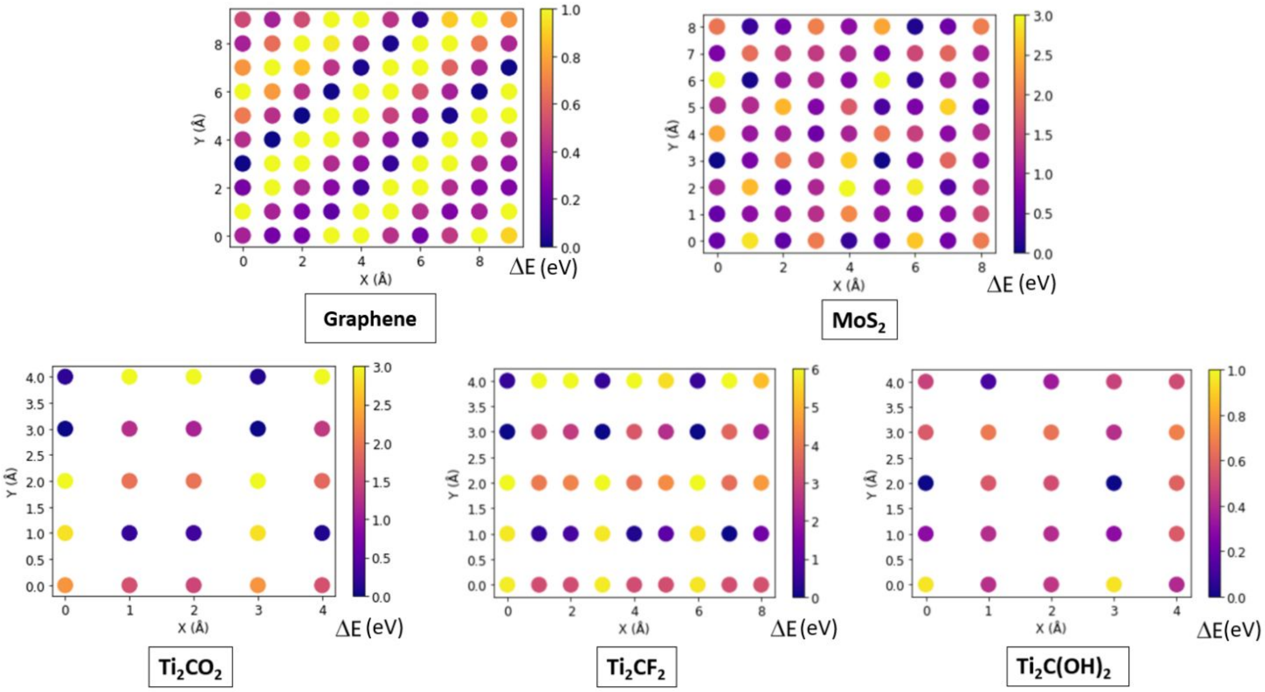

Figure S9. Potential energy surface of rigid adsorption of MXene, MoS_2, and graphene on Hematite (001) surface.

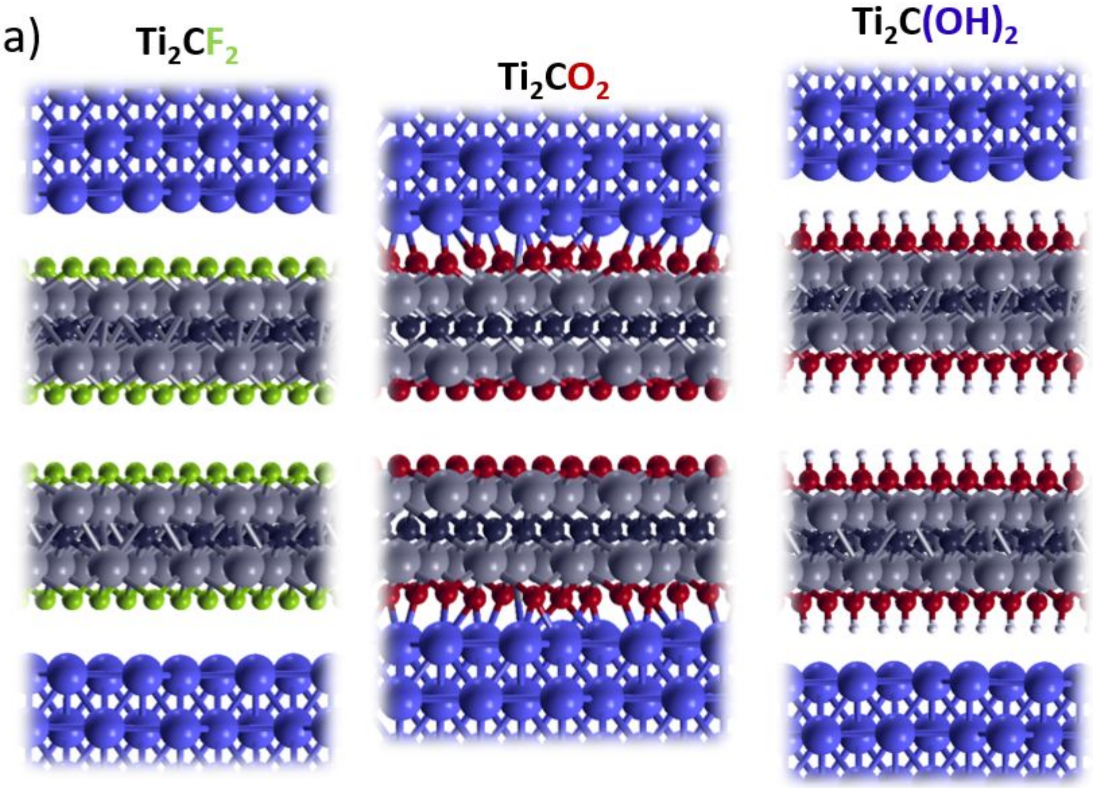

b)

	Ti_2CF_2	Ti_2CO_2	$Ti_2C(OH)_2$
$W_{SEP\ within\ iron}$	-0.15	-0.16	-0.14
$W_{SEP\ free}$	-0.10	-0.10	-0.14

Figure S10. a) Iron interfaces fully covered by MXene layers. The models were created by the symmetric reflection of relaxed MXene-on-Fe structures. b) W_{SEP} (in J/m^2) calculated for both MXenes within the Fe substrate and free. The considered lateral positions correspond to terminations pointing against each other, i.e., PES maxima.
